# Psychological and anthropometric outcomes in preschool children exposed to antenatal maternal obesity: modelling of early life risk factors

**DOI:** 10.1136/bmjment-2026-302738

**Published:** 2026-10-12

**Authors:** Julie Sigurdardottir, Kathryn Dalrymple, Mary Rutherford, Lucilla Poston, on behalf of the UPBEAT Consortium, Lucilla Poston

**Affiliations:** 1 Department of Forensic and Neurodevelopmental Sciences, Institute of Psychiatry, Psychology and Neuroscience, King’s College London, London, UK; 2 Department of Nutritional Sciences, School of Life Course and Population Health Sciences, King’s College London, London, UK; 3 Research Department of Early Life Imaging, School of Biomedical Engineering and Imaging Sciences, King’s College London, London, UK; 4 Department of Women and Children’s Health, School of Life Course and Population Health Sciences, King’s College London, London, UK; 5 King’s College London, London, UK

**Keywords:** Attention Deficit and Disruptive Behavior Disorders, Child Behavior Disorders, Feeding and Eating Disorders of Childhood, Psychometrics, Psychopathology

## Abstract

**Background:**

Maternal obesity is associated with increased risk of adverse neurodevelopmental outcomes and obesity in offspring, yet their comorbidity among preschoolers and underlying mechanisms remain unclear.

**Objective:**

To examine associations between maternal obesity in pregnancy and offspring preschool psychopathology, focusing on clinical and biological pathways.

**Methods:**

In a cohort of 462 women with obesity in pregnancy (body mass index (BMI) ≥30 kg/m²) from the UK Pregnancies Better Eating and Activity Trial, followed up with their children at 3–4 years postpartum (49% boys), structural equation models assessed effects of antenatal exposures (measured at three visits) on child risk of psychopathology: maternal inflammation (by interleukin-6), glycaemia (by random glucose or gestational diabetes diagnosis), placental function (by placental growth factor) and depressive symptoms. Child outcomes were derived from the Strengths and Difficulties Questionnaire scores and psychological phenotypes from latent class analysis (LCA). Child adiposity and eating behaviours were compared across phenotypes.

**Findings:**

High-risk of psychopathology was identified in 11.3% of children (boys 14.7%, girls 8%). Higher antenatal maternal depression and lower placental function predicted increased risk of psychopathology, while inflammation and glycaemia were not associated (R^2^=44.5%). LCA identified externalising (8%) and internalising (4.1%) phenotypes. An externalising phenotype was associated with higher BMI, weight-for-age z-scores and waist circumference; both phenotypes showed greater food fussiness than well-adjusted children.

**Conclusions:**

One in 10 children of women with obesity showed early risk of psychopathology, which was linked to adiposity. Antenatal maternal depression emerged as the primary risk factor, with a novel role for placental dysfunction.

**Clinical implication:**

Screening for maternal depression and placental function in pregnancies affected by obesity may help reduce adverse neurodevelopmental and metabolic outcomes in offspring.

**WHAT IS ALREADY KNOWN ON THIS TOPIC**

- Maternal obesity in pregnancy increases likelihood of offspring neurodevelopmental diagnoses and obesity. Maternal hyperglycaemia, depression and inflammation have been implicated in the causal pathways but seldom jointly with obesity, along other biological markers such as placental function.

**WHAT THIS STUDY ADDS**

- Structural equation models provide a novel approach to clarify which facet(s) of obesity in pregnancy significantly contribute to adverse outcomes in the child.
- Not only was antenatal maternal depression a contributor to preschool child psychological outcomes, we also found a significant role of placental function and clarified that maternal inflammation and glycaemic level were unlikely associated. Our final model explained 44.5% of the outcome variance.
- We show that the comorbidity of externalising behaviour with increased adiposity and fussy eating behaviour is present as early as preschool age.

**HOW THIS STUDY MIGHT AFFECT RESEARCH, PRACTICE OR POLICY**

- Future research should consider that mental and cardiovascular health risks in childhood are intertwined and should not be studied independently.
- Monitoring maternal antenatal depression and placental function in the pregnant population with obesity may be targets for intervention towards reducing adverse transgenerational health outcomes in a global population showing increasing rates of obesity and psychiatric diagnoses.

## Background

 In 2021 almost half of the adult world population was overweight or obese, including approximately 1.11 billion women.^1^ Compared with women with body mass index (BMI) in the normal range (18.5–24.9 kg/m^2^), when pregnant, women living with obesity have heightened risk of gestational diabetes (GDM)^2^ and complications in labour and postpartum^3^; a higher prevalence of maternal depression is also reported among women living with obesity when compared with women of normal BMI.^4^ The infant is at greater risk of macrosomia, stillbirth and neonatal death but health in the longer term may also be compromised.^5^ Studies addressing psychological outcomes in children exposed to maternal obesity in utero report greater likelihood of autism, attention-deficit hyperactivity disorder (ADHD), poorer cognitive outcomes and a potential compounding effect of comorbid maternal hyperglycaemia.^6–9^ A higher incidence of obesity and adulthood type-2 diabetes and cardiovascular disease in these children is also well documented.^10 11^ Importantly, there is increasing evidence for a relationship between childhood autism and/or ADHD diagnosis and overweight/obesity among children,^12–16^ as well as abnormal eating behaviours.^17^

While shared genetic susceptibility between maternal obesity and offspring adiposity and neuropsychological outcomes remains a possibility, the current preclinical and human evidence base suggest key roles of in utero exposures. Nevertheless, previous attempts to define compounding maternal exposures in women with obesity, which may have persistent effect the developing infant brain, have focused primarily on only one antenatal/perinatal exposure, for example, maternal infection or a low-quality diet, mental health or hyperglycaemia, in samples of mixed BMI and using single-timepoint antenatal measures of the exposures.^18–24^ Furthermore, confounding or additive roles for maternal psychopathology, inflammatory processes and placental function in those explorations are seldom considered.^25–28^ We previously showed in this cohort that maternal antenatal depression was more likely to be accompanied by a combination of raised blood glucose, inflammation and lower placental function, as measured by placental growth factor.^29^ A predisposition to psychopathology in early life via these exposures, and their likely interplay, is supported in the literature. Disruptions in the metabolic and inflammatory responses of the placenta can lead to suboptimal fetal supply of nutrients, oxygen and endocrine signalling.^27 28^ Downstream effects include disrupted development in the fetal stress regulatory pathway, especially in raised maternal cortisol exposure associated with poor mental health.^20^ Additionally, under homeostasis, insulin and many inflammatory cytokines are key regulators for neuronal development and synaptogenesis. However, the fetal hyperinsulinaemic response to maternal hyperglycaemia or a heightened cytokine influx through the placenta have shown to change neurodevelopmental trajectories.^25^

## Objectives

We propose that a holistic theoretical and statistical approach is required to study the health of children exposed to antenatal obesity and thereby the risk factors, potentially modifiable by intervention.^30^ In this study we describe outcomes among preschoolers born to mothers with obesity who participated in the UK Pregnancies Better Eating and Activity Trial (UPBEAT).^31^ Capitalising on the available repeated clinical and biological measures in pregnancy, we aimed to tease apart the relative contribution of the co-occurring maternal exposures mentioned above towards child risk of psychopathology through successive theoretically driven structural equation models (SEMs). To date, interactions between psychological outcomes, adiposity and related eating behaviours have not been studied together in preschool children born of women with obesity. A second aim was therefore to employ latent class analysis (LCA)^32^ to explore further heterogeneity in child phenotypes using a data-driven person-oriented mixture modelling approach. By developing models of predisposition to psychopathology via antenatal exposures and a child trait-centred approach to health profiling at a critical stage of development, we aim to inform strategies for early intervention.

## Methods

### Participants

The UPBEAT^31^ originally comprised 1555 pregnant women without pre-existing diabetes recruited between March 2009 and June 2014. The study was a randomised controlled trial of a lifestyle intervention which did not demonstrate any change in the primary outcomes; GDM or delivery of a large for gestational age (LGA) infant. Extensive demographic, anthropometric, pregnancy, blood and birth outcomes were collected through UPBEAT at enrolment, study visits in pregnancies (17 weeks (baseline, visit 1), 27 weeks (visit 2) and 34 weeks gestation (visit 3)) and from electronic medical records. All participants were invited for a follow-up 3–4 years after delivery and a total of 514 mother–child dyads had accepted to be followed-up at 3–4 years after birth and; 462 children were included after following the application of exclusion criteria: missing primary measure of psychological outcome (Strengths and Difficulties Questionnaire (SDQ),^33^) delivery <34 weeks gestation, chromosomal or congenital abnormality, or maternal pre-eclampsia.

Compared with the original UPBEAT cohort (online supplemental table S1), the 462 mothers who attended the 3–4-year visit were of a lower BMI, older, more likely of white ethnicity, earning higher income, nulliparous, persons who do not smoke and of higher education. A lower proportion were in the most deprived quintile of the Index of Multiple Deprivation. There were no differences in proportion assigned to the intervention or standard care arms of the trial, or in GDM diagnosis or depressive symptoms scores in the antenatal period. Compared with the original cohort, the children included were born overall at higher gestational age and higher WHO birth weight centile (online supplemental table S2). To improve power, the SEMs included all available antenatal outcome data of the original cohort following the same above exclusion criteria (total n=1357, see online supplemental figure S2).

### Maternal in utero risk factors for child psychopathology

The SEMs were comprised of latent factors for maternal *glycaemia*, *depression*, *placental function*, *inflammation* or *obesity*. Latent factorisation using repeated measures combines their joint measurement distributions, minimising the nuisance of time on their accuracy and accounts better for the measurement error otherwise introduced when using proxies. This approach enabled the improvement of regression estimates, when effect sizes are likely small and when the measures are unstable over time.^34^ SEM also tests model fit of complex relationships between maternal health and offspring outcomes. Online supplemental table S3 indicates that the data showed minimal missingness, which was dealt with our statistical approach under missing-at-random (see section ‘Pathway modelling with SEMs’). Blood samples were collected from six of the eight UPBEAT trial centres (for logistical reasons), hence 94 women (20.3%) had no blood biomarkers.

Model 1 includes the GDM diagnosis, which is replaced by a latent *glycaemia* factor for comparison in Models 2, 3 and 4. GDM was diagnosed at 24–28 weeks gestation (visit 2) according to the International Association of Diabetes and Pregnancy Study Groups guidelines: fasting glucose ≥5.1 mmol/L and/or 1-hour glucose ≥10 mm/L and/or 2-hour glucose ≥8.5 mmol/L. *Glycaemia* was indicated by the random plasma glucose measures available at visit 1 and 3. *Placental function* was indicated by placental growth factor (PlGF) collected at visit 1 and 2. PlGF is a biomarker of placental trophoblast function and angiogenesis and a surrogate for maternal to fetal nutrient/metabolite transfer capacity. Latent *inflammation* was estimated by interleukin-6 (log IL-6) at visits 1 and 2. Blood collection and analysis was described previously.^29^ *Antenatal depression* was indicated by the Edinburgh Postnatal Depression Scale (EPDS) sum score at three visits.^35^ Maternal *obesity* was indicated by maternal baseline BMI, sum of skinfold thicknesses, waist and hip circumferences (all continuous).

The maternal EPDS score at the 3–4-year follow-up was included to control for effects of maternal depressive symptoms on (1) her perception and reporting of difficulties in the child and (2) parent–child attachment in the causal pathways to the child’s neurodevelopment and behaviour. The regression path from maternal antenatal depression in pregnancy to postnatal depression was also included. We included child sex assigned at birth and gestational age at birth in all models.

### Child outcome variables

#### Primary outcome

##### The Strengths and Difficulties Questionnaire

Parents/carers completed the SDQ for the 3–4 years age-range (www.sdqinfo.org). The SDQ^36^ comprises 25 items divided equally into five subscales each scoring 1–5 points: emotional symptoms (*emotion*), conduct problems (*conduct*), hyperactivity/inattention (*hyperactive*), peer relationship problems (*peer*) and prosocial behaviour (*prosocial*). The total SDQ score provides an overall difficulty score, derived from summing the first four scales (0–40 points). *Conduct* and *hyperactivity* have been used in conjunction to assess *externalising* problems and the *emotion* and *peer* scales have been associated with *internalising* problems.^37^ We excluded the *prosocial* subscale from phenotype analyses with LCA as we focused on exploring risks to deficits in emotion regulation and to challenging behaviours, reflecting thus the SDQ total score calculation. Total SDQ and subscale scores were first dichotomised as high/low risks according to reported cut-offs for 3–4 years old children (www.sdqinfo.org) to ease comparisons with existing literature and for the LCA as follows: *Total SDQ≥16; emotional problems≥4; conduct problems≥5; hyperactivity≥7; peer problems≥4*. The SDQ reported by carers or teachers has shown satisfactory psychometric properties, including when administered to British preschoolers.^38–40^

### Secondary outcomes

#### Adiposity and BMI

Child anthropometric measurements for estimation of adiposity included waist and arm circumferences, sum of skinfold thicknesses and z-scores of height-for-age, weight-for-age and BMI, calculated according to the WHO Child Growth Standards for each sex as previously described.^41^ All thicknesses and circumferences were measured in triplicate by trained research staff and the average generated.

#### Child Eating Behaviour Questionnaire

The Child Eating Behaviour Questionnaire is a validated parent-reported method to assess child’s eating style, including food avoidance and food approach behaviours.^42^ The 35-item questionnaire is divided into eight eating behaviours (food-responsiveness, emotional overeating, enjoyment of food, desire to drink, satiety responsiveness, slowness in eating, emotional undereating and food fussiness). Each question is rated on a 5-point Likert scale (Never=1, Always=5). Higher scores indicate a greater tendency towards the corresponding behaviour. For example, the food fussiness subscale captures selective eating behaviours, including reluctance to try new foods, limited acceptance of a variety of foods, difficulty being pleased with meals and rejection of foods before tasting them. Whereas, enjoyment of food is characterised by children who love food and look forward to meal times.

### Statistical analysis

#### Group comparisons

To compare and provide reference with previous relevant literature, univariate group-wise comparisons are reported for child psychological and anthropometric outcomes and eating behaviour. Pregnancy, birth events and antenatal maternal factors were also compared by child sex, in order to assess potential discordant exposures for subsequent pathway modelling. In R, t-tests were used for normally distributed continuous variables and Kruskal-Wallis test for non-parametric variables and dichotomous/categorical variables were compared by χ² tests.

#### Pathway modelling with SEMs

Four SEMs models were tested iteratively from simple to more complex (figure 1A and online supplemental figure S2), following published methods^43^ and^44^ were tested in *Mplus*.^45^ Here, the High/Low risk status (dichotomised) based on the overall difficulty total SDQ score was the child outcome variable. Regression paths included those identified in the literature, for example, higher *obesity* predicts higher risks of GDM diagnosis/hyperglycaemia. The trial intervention was not included as it did not impact primary maternal (diabetes) and child outcomes (LGA) and our variables did not differ between control and intervention arms previously.^31 41^ Additionally, we found no difference in proportion of high-risk versus low-risk children in terms of their maternal randomised allocation. The Bayes estimator was used throughout (over frequentist approach using maximum likelihood) to improve numerical integration and obtain convergence as SEM complexity increases. Here, the standardised regression/covariance coefficient for direct (blue lines on figure 1A) and indirect paths (green lines) are reported and the 95% *credibility intervals* (CIs) excluding zero is interpreted as significant for the coefficient. The Bayesian estimation handles missing data under the missing-at-random data theory,^46 47^ allowing all available data to be included in the analyses. Further details on SEM specification are in the online supplemental information (page 9).

**Figure 1:**
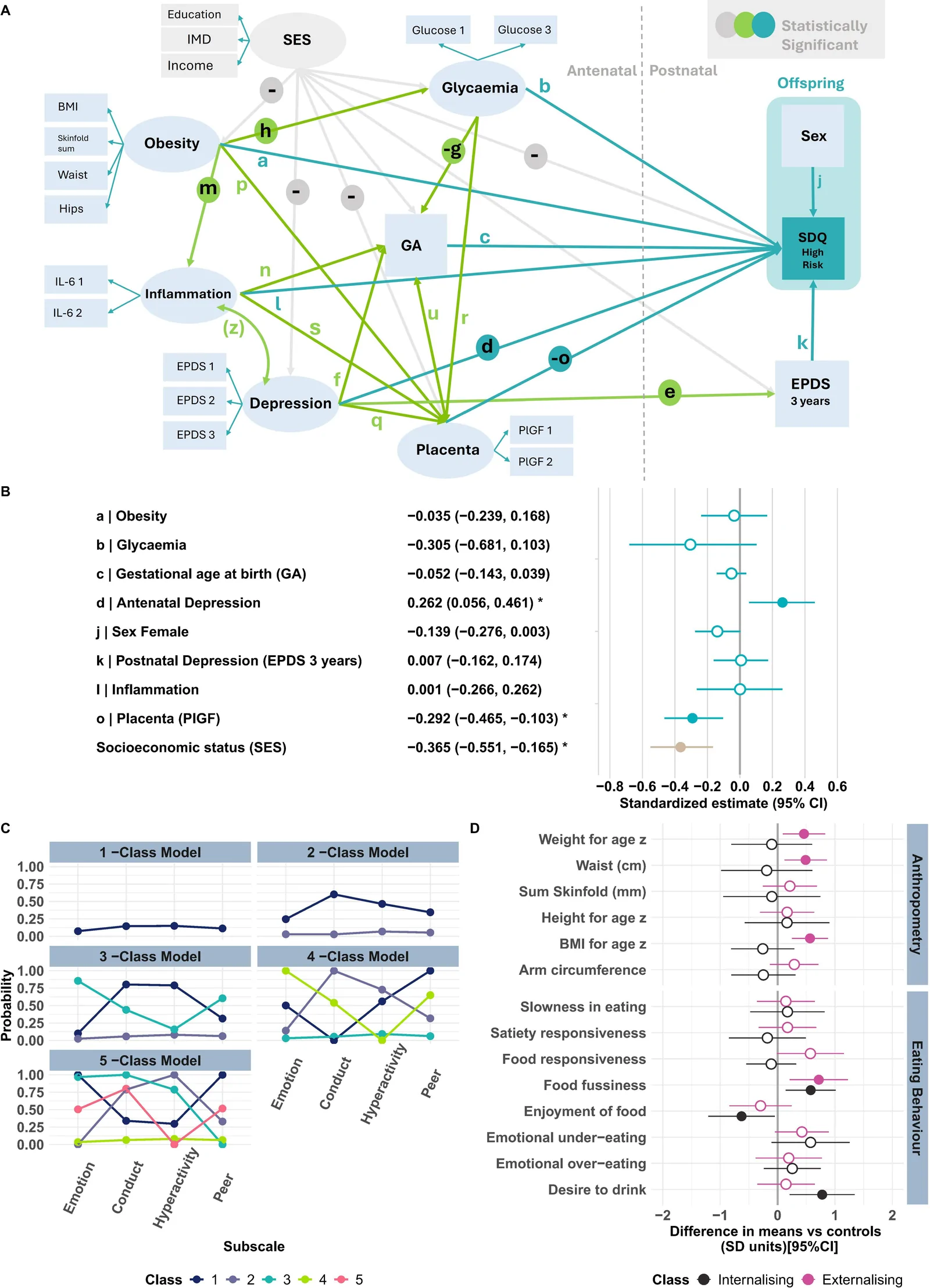
Final SEM, child psychological phenotyping and outcomes. (A) SEM with main direct effect paths of interest to high-risk of psychopathology (SDQ total score ≥16) in blue and other indirect paths included in green. Visit 1 occurred at median 17 weeks (study baseline), visit 2 at 27 weeks and visit 3 at 34 weeks gestation. Latent variables are depicted as ovals and observed variables as squares. All latent variables indicators loaded such that, for example, higher *SES*=better, higher *depression*=more symptoms. Curved bidirectional path refers to a correlation. Letters refer to the beta coefficients labels reported in table 2 and the sign denotes the direction of the effect. Residuals are not shown. (B) Direct standardised effects and 95% credibility intervals on high-risk of psychopathology in the offspring, derived from the SEM in A. For complete details see table 2. (C) LCA was performed to uncover psychological phenotypes based on the SDQ subscales (x-axis). The 3-class model was accepted for further analyses. (D) Based on the 3-class LCA model, the standardised mean scores of the ‘externalising’ group and ‘internalising’ group of children were compared with the “well-adjusted” group of children on anthropometry and eating behaviours, including sex as a covariate where appropriate. SEM available data: BMI=1357, sum of skinfold=1338, hip circumference=1346, waist circumference=1346, EPDS 1=1181, EPDS 2=1056, EPDS 3=932, GA=1357, GDM=270 diagnosed and 887 not diagnosed, Glucose 1=948, Glucose 3=735, High SDQ=52 yes and 410 no, IL-6 1=905, IL-6 2=825, child sex=1357, PlGF 1=958, PlGF 2=876. BMI, body mass index; EPDS, Edinburgh Postnatal Depression Scale; GDM, gestational diabetes; IL, interleukin; IMD, Index of Multiple Deprivation; PlGF, placental growth factor; SDQ, Strengths and Difficulties Questionnaire; SEM, structural equation model.

#### Latent class analysis

The presence of latent subgroups/classes based on the SDQ subscales was probed on the assumption that some children may present as primarily at risk of *internalising* or *externalising* problems or could possibly show widescale psychological and behavioural issues. In *Mplus 8.3*, 1–5 class LCA models were run using reported cut-off scores (low vs high-risk, coded 0/1), for the whole sample first and then for each sex separately. Details on model estimation, fit and selection criteria are found in the online supplemental information (page 11). Following the LCA, the probabilities of being male or female in each class were calculated (*DCAT* in *Mplus*^48 49^). Latent classes were compared on anthropometric measures and eating behaviour using the *BCH 3-step* approach^49^ including child sex as a covariate where appropriate. Both DCAT and BCH take into account the fractional membership error of the LCA.

The *Mplus* outputs were integrated into R package *MplusAutomation*^50^ and the figures generated with the *ggplot2*.

## Findings

The sample comprised 462 mothers of mean (SD) 32.29 (5.29) years of age, 68.8% were white, 22.3% black, 40.2% pertained to the most deprived quintile of the Index of Multiple Deprivation. For 43.7%, their income was below £32 500/year and 44.8% had a university degree. We found 52/462 (11.3%) of children scored equal or above the 16-point threshold on the SDQ and their mothers were less likely to have a university degree (p=0.027) and earning above £32 500/year (p=0.002) than mothers whose children scored lower than 16, see table 1 .

**Table 1:** Maternal baseline characteristics by child risk of psychopathology

	Total	Low-risk SDQ<16	High-risk SDQ≥16	P value
n	462	410	52	
BMI (median (IQR))	34.70 (32.60–37.80)	34.70 (32.60–37.77)	34.15 (32.45–38.20)	0.938
Age (years) (mean (SD))	32.29 (5.29)	32.49 (5.18)	30.72 (5.96)	0.023
Main ethnicity (%)				
White	318 (68.8)	286 (69.8)	32 (61.5)	0.553
Black	103 (22.3)	90 (22.0)	13 (25.0)	
Asian	19 (4.1)	16 (3.9)	3 (5.8)	
Other	22 (4.8)	18 (4.4)	4 (7.7)	
IMD (%)				
Least deprived	27 (5.9)	26 (6.4)	1 (1.9)	0.243
Second quintile	32 (7.0)	30 (7.4)	2 (3.8)	
Third quintile	51 (11.1)	47 (11.5)	4 (7.7)	
Fourth quintile	165 (35.9)	140 (34.3)	25 (48.1)	
Most deprived	185 (40.2)	165 (40.4)	20 (38.5)	
Income (%)				
<£17 629	108 (23.4)	90 (22.0)	18 (34.6)	0.002
£17 629–32 500	94 (20.3)	81 (19.8)	13 (25.0)	
>£32 500	208 (45.0)	197 (48.0)	11 (21.2)	
Prefers not to answer	52 (11.3)	42 (10.2)	10 (19.2)	
Highest education (%)				
None/GCSE	76 (16.5)	62 (15.1)	14 (26.9)	0.027
Vocational qualification	113 (24.5)	96 (23.4)	17 (32.7)	
A level (or equivalent)	66 (14.3)	60 (14.6)	6 (11.5)	
University	207 (44.8)	192 (46.8)	15 (28.8)	
Born in the UK (%)	328 (71.0)	293 (71.5)	35 (67.3)	0.646
Nulliparous (%)	223 (48.3)	196 (47.8)	27 (51.9)	0.680
Smoking (%)				
Never	335 (72.5)	300 (73.2)	35 (67.3)	0.412
Current	19 (4.1)	18 (4.4)	1 (1.9)	
Ex—gave up before pregnancy	69 (14.9)	60 (14.6)	9 (17.3)	
Ex—gave up in pregnancy	39 (8.4)	32 (7.8)	7 (13.5)	
Intervention (%)	227 (49.1)	199 (48.5)	28 (53.8)	0.566

Note: Baseline variables at antenatal trial entry of 462 mothers attending the 3–4-year follow-up visit and split by child overall risk of psychopathology from the SDQ. Normally and non-normally distributed variables are compared with a t-test and a Kruskal-Wallis test, respectively. Categorical variables were analysed with a χ² test.

BMI, body mass index; GCSE, General Certificate of Secondary Education; IMD, Index of Multiple Deprivation; SDQ, Strengths and Difficulties Questionnaire.

### Structural equation models to risk of psychopathology

Four SEMs were specified (online supplemental figure S2). Models 1–4 reached convergence and were close to or below a PSR of 1.1. The Posterior predicted p values were above >0.05. Details on the model convergence and fit, including trace plots, are found in the online supplemental information table S9 and figure S4.

The standardised beta coefficients are in table 2. In *Model 1*, the main effect of latent antenatal depression was the only direct significant and positive effect on child high-risk of psychopathology (standardised beta 95% CI 0.29 (0.10 to 0.47)). The main direct effects of latent maternal *obesity*, maternal GDM, gestational age at birth, female sex and maternal 3–4 year EPDS score on child high-risks of psychopathology were all non-significant. Antenatal maternal *depression* predicted lower GA at birth (−0.09 (−0.15 to −0.03)) and higher maternal EPDS score at the follow-up (0.59 (0.51 to 0.65)). GDM predicted earlier GA at birth (−0.37 (−0.44 to −0.30)) and was in turn predicted by latent *obesity* (0.14 (0.06 to 0.21)). Other paths were not significant. *Model 2* suggests latent maternal *glycaemia* did not directly predict child high risks of psychopathology and the same significant effects of maternal antenatal *depression* on child high-risk of psychopathology remained. In this model female sex was negatively associated with these outcomes (−0.15 (−0.29 to −0.004)).

**Table 2:** Standardised coefficients (95% CI) from SEM regression model paths

	Direct	Indirect	Model 1 GDM	Model 2 glycaemia	Model 3 inflammation	Model 4 placenta	Model 4 SES-adjusted
*a*	Obesity		−0.034 (−0.200 to 0.125)	0.024 (−0.159 to 0.216)	−0.004 (−0.213 to 0.208)	−0.002 (−0.210 to 0.200)	−0.035 (−0.239 to 0.168)
*b*	GDM/glycaemia		−0.107 (−0.328 to 0.123)	−0.318 (−0.563 to 0.502)	−0.331 (−0.564 to 0.667)	−0.333 (−0.687 to 0.045)	−0.305 (−0.681 to 0.103)
*c*	GA		−0.056 (−0.161 to 0.059)	−0.067 (−0.169 to 0.035)	−0.064 (−0.165 to 0.036)	−0.063 (−0.157 to 0.034)	−0.052 (−0.143 to 0.039)
*d*	AD		0.290 (0.098 to 0.468)*	0.309 (0.118 to 0.486)*	0.307 (0.114 to 0.484)*	0.324 (0.135 to 0.497)*	0.262 (0.056 to 0.461)*
*e*		AD → Postnatal depression	0.586 (0.512 to 0.652)*	0.586 (0.510 to 0.651)*	0.586 (0.510 to 0.651)*	0.586 (0.511 to 0.652)*	0.581 (0.501 to 0.651)*
*f*		AD → GA	−0.087 (−0.145 to −0.028)*	−0.086 (−0.146 to −0.026)*	−0.084 (−0.144 to −0.023)*	−0.084 (−0.144 to −0.023)*	−0.057 (−0.125 to 0.014)
*g*		GDM/glycaemia → GA	−0.370 (−0.436 to −0.300)*	**−0.226 (−0.348 to 0.125**)	**−0.211 (−0.337 to 0.191**)	−0.230 (−0.357 to −0.107)*	−0.222 (−0.353 to −0.100)*
*h*		Obesity → GDM/glycaemia	0.136 (0.059 to 0.211)*	0.188 (−0.114 to 0.303)	0.178 (−0.197 to 0.297)	0.186 (0.069 to 0.306)*	0.177 (0.058 to 0.298)*
*i*		Obesity → GA	0.001 (−0.054 to 0.057)				
*j*	Female offspring		−0.142 (−0.284 to 0.005)	−0.148 (−0.288 to −0.004)*	−0.146 (−0.284 to −0.001)*	−0.116 (−0.256 to 0.029)	−0.139 (−0.276 to 0.003)
*k*	Postnatal depression		0.033 (−0.144 to 0.206)	0.020 (−0.156 to 0.193)	0.019 (−0.155 to 0.191)	0.003 (−0.169 to 0.172)	0.007 (−0.162 to 0.174)
(*x*)		Obesity ↔ AD	0.024 (−0.038 to 0.085)				
*l*	Inflammation				0.081 (−0.185 to 0.344)	0.056 (−0.216 to 0.311)	0.001 (−0.266 to 0.262)
*m*		Obesity → Inflammation			0.388 (0.302 to 0.471)*	0.390 (0.304 to 0.474)*	0.391 (0.304 to 0.475)*
*n*		Inflammation → GA			−0.039 (−0.123 to 0.047)	−0.035 (−0.121 to 0.051)	−0.031 (−0.117 to 0.056)
(*z*)		Inflammation ↔ AD			0.069 (−0.037 to 0.174)	0.070 (−0.036 to 0.175)	
*o*	Placenta					−0.216 (−0.385 to −0.033)*	−0.292 (−0.465 to −0.103)*
*p*		Obesity → Placenta				−0.060 (−0.151 to 0.027)	−0.073 (−0.163 to 0.015)
*q*		AD → Placenta				−0.005 (−0.091 to 0.077)	−0.056 (−0.147 to 0.031)
*r*		Glycaemia → Placenta				0.108 (−0.025 to 0.247)	0.089 (−0.051 to 0.237)
*s*		Inflammation → Placenta				0.001 (−0.115 to 0.114)	−0.019 (−0.136 to 0.095)
*t*		Female → Placenta				−0.001 (−0.072 to 0.068)	
*u*		Placenta → GA					−0.039 (−0.115 to 0.042)
	SES						−0.365 (−0.551 to −0.165)*
		SES → Obesity					−0.119 (−0.185 to −0.053)*
		SES → AD					−0.181 (−0.254 to −0.106)*
		SES → Glycaemia					−0.037 (−0.186 to 0.110)
		SES → Placenta					−0.207 (−0.299 to −0.116)*
		SES → GA					0.049 (−0.026 to 0.124)

Note: The letters in the first column refer to the paths labels in the models (figure 1A and online supplemental figure S2Supplemental Figure S2), where offspring high-risk for psychopathology from the Strengths and Difficulties Questionnaire is the dependent dichotomised variable. All paths are from Bayesian statistics regressions estimations, the letters inside parentheses are correlations. Maternal antenatal obesity, antenatal inflammation, AD, antenatal glycaemia and placental function are latent factors.

* in bold= Significance defined as estimate 95% CIs do not include 0.

AD, antenatal depression; GA, gestational age at birth; GDM, gestational diabetes; SEM, structural equation model; SES, socioeconomic status.

Model 3 shows antenatal *inflammation* had no direct effect on the child outcomes, while the direct effect of antenatal *depression* remained. The effect of latent antenatal *obesity* on latent antenatal *inflammation* was positive (0.39 (0.30 to 0.47)).

*Model 4* shows higher antenatal *depression* (0.32 (0.14 to 0.50)) and lower *placental function* (−0.22 (−0.39 to −0.03)) associated directly with higher child risk of psychopathology. To remove potential confounding effects of socioeconomic status (SES), we repeated Model 4 post hoc with child outcomes and exposures also adjusted for maternal *latent SES* (SES indicated by Education, Index of Deprivation and Income). The significant results for *depression* and *placental function* remained (0.26 (0.06 to 0.46) and −0.29 (−0.47 to −0.10), respectively) and better *SES* was a significant negative predictor of high-risk outcome (−0.37 (−0.55 to −0.17)). See figure 1B. Mplus outputs for Model 4 and SES-adjusted Model 4 are shown in the online supplemental information (page 14–19). As a sensitivity analysis, we reran the final SES-adjusted Model 4 using the total continuous SDQ score as the outcome measure. Antenatal *depression* effect remained significant (0.19 (0.06 to 0.32]) and so did the *placenta* effect (−0.15 [−0.26 to −0.04]).

Model 1 explained 16% of the variance in the high-risk child psychological outcome and in Model 2, changing GDM for *glycaemia*, R^2^ reached 26%. Adding latent inflammation (Model 3) increased R^2^ to 29%, with *placental function* (Model 4) to 34.6%. Model 4 *SES*-adjusted was 44.5%. Using the continuous SDQ score instead as the outcome, the total variance explained was 19.8%.

### SDQ and adiposity

At mean age 3.5 (0.3) years and based on reported SDQ cut-off scores, 52 of 462 children (11.3%) were considered at *overall* high risk of psychopathology. Distribution of child age at visit, SDQ scores and anthropometry are presented in online supplemental figure S1A,C,D. The subscale scores were moderately correlated with each other but not with child age at the assessment (online supplemental figure S1B). Mothers of children who were considered high-risk on the Overall SDQ score had lower education and income and were younger than mothers of low-risk children (table 1). Furthermore, 14.5% of children were considered at high risk of *conduct* problems and 14.9% of *hyperactivity*, 7.4% and 11.3% were high-risk on the *emotion* and the *peer* problems subscales, respectively. The mean (SD) WHO z-scores of 0.87 (1.04) for BMI, 0.39 (1.10) for height and 0.83 (1.06) for weight indicated this sample had a larger morphology on average than the WHO standards for this age (table 3).

**Table 3:** Child outcomes at 3–4 year follow-up by sex

	Overall	Male	Female	P value
n	462	225	237	
Age (months)	41.75 (3.40)	41.89 (3.36)	41.61 (3.44)	0.367
SDQ				
SDQ total score	8.00 (5.00–12.00)	9.00 (6.00–13.00)	7.00 (5.00–11.00)	0.002
SDQ≥16 (%)	52 (11.3)	33 (14.7)	19 (8.0)	0.035
Emotion total	1.00 (0.00–2.00)	1.00 (0.00–2.00)	1.00 (0.00–2.00)	0.796
Emotion≥4 (%)	34 (7.4)	14 (6.2)	20 (8.4)	0.463
Conduct total	2.00 (1.00–4.00)	2.00 (1.00–4.00)	2.00 (1.00–3.00)	0.016
Conduct≥5 (%)	67 (14.5)	42 (18.7)	25 (10.5)	0.019
Hyperactive total	4.00 (2.00–5.00)	4.00 (2.00–6.00)	3.00 (2.00–5.00)	<0.001
Hyperactive≥7 (%)	69 (14.9)	44 (19.6)	25 (10.5)	0.010
Peer total	1.00 (0.00–2.00)	1.00 (0.00–2.00)	1.00 (0.00–2.00)	0.040
Peer≥4 (%)	52 (11.3)	30 (13.3)	22 (9.3)	0.219
Anthropometry				
BMI for age z-score	0.87 (1.04)	0.90 (0.97)	0.85 (1.11)	0.617
Height for age z-score	0.39 (1.10)	0.62 (1.02)	0.18 (1.13)	<0.001
Weight for age z-score	0.83 (1.06)	0.99 (0.90)	0.68 (1.18)	0.002
Waist (cm)	52.96 (4.23)	52.74 (4.13)	53.17 (4.33)	0.294
Sum of skinfold (mm)	41.30 (33.98–50.32)	38.45 (32.50–46.30)	44.00 (35.32–53.40)	<0.001
Arm circumference	17.72 (1.69)	17.63 (1.49)	17.81 (1.86)	0.289
Eating behaviour				
Food responsiveness	2.00 (1.40–2.60)	2.00 (1.40–2.70)	2.00 (1.40–2.60)	0.996
Satiety responsiveness	3.11 (0.75)	3.08 (0.71)	3.14 (0.79)	0.354
Emotional under-eating	2.65 (0.96)	2.72 (1.00)	2.58 (0.91)	0.128
Emotional overeating	1.25 (1.00–1.75)	1.25 (1.00–1.75)	1.25 (1.00–1.75)	0.441
Food fussiness	2.91 (0.95)	2.98 (0.93)	2.85 (0.97)	0.135
Slowness in eating	3.13 (0.87)	3.11 (0.86)	3.15 (0.88)	0.666
Enjoyment of food	3.56 (0.91)	3.53 (0.92)	3.58 (0.90)	0.504
Desire to drink	2.78 (1.21)	2.82 (1.24)	2.74 (1.18)	0.509

Note: Normally and non-normally distributed variables are presented as means(SD) and median (IQR) and compared with a t-test and a Kruskal-Wallis test, respectively. All z-scores are according to WHO standards for each sex adjusted for age. Categorical variables are shown as counts (%) and analysed with a χ² test.

BMI, body mass index; SDQ, Strengths and Difficulties Questionnaire.

A larger proportion of males than females were *overall* high-risk of psychopathology (total SDQ, 14.7% vs 8%, p=0.035), including *conduct* problems (18.7% vs 10.5%, p=0.019) and *hyperactivity* (19.6% vs 10.5%, p=0.010) but proportions on the *emotion* problems (6.2% vs 8.4%) and *peer* problems (13.3% vs 9.3%) were not significantly different. Males’ height-for-age z-score was higher than girls’ at the follow-up (p<0.001) but there were no differences in BMI-for-age, arm or waist circumferences. Females had higher mean sum of skinfold thicknesses than males (p<0.001). There were no differences in eating behaviour between the sexes, see table 3. In birth outcomes, male birth weight was expectedly higher and they were more often delivered by C-section than girls, see online supplemental table S4.

### Associations between psychological outcome, body morphology and eating behaviour

Boys who are at high-risk of psychopathology (>16 points) compared with low-risk boys were of higher BMI (z-score: 1.27 (0.80) vs 0.84 (0.98), p=0.024), higher weight z-score (1.34 (0.98) vs 0.93 (0.87), p=0.017), larger waist (54.81 cm (4.05) vs 52.43 cm (4.06), p=0.004) and arm circumferences (17.54 cm (1.45) vs 18.19 cm (1.61), p=0.031). High-risk boys showed higher food-fussiness (2.90 (0.91) vs 3.46 (0.90), p=0.002). There were no differences in anthropometry between high-risk and low-risk girls but high-risk girls also presented higher food-fussiness (2.81 (0.94) vs 3.30 (1.20), p=0.035), enjoyed food less (3.63 (0.87) vs 3.00 (1.07), p=0.003) and showed higher emotional undereating (2.54 (0.89) vs 3.12 (1.02), p=0.008). There were no differences in birth outcomes between high-risk and low-risk children which may influence later adiposity, such as LGA or birth weight. See online supplemental table S5.

### Psychological phenotyping by latent class analysis

Online supplemental table S6 provides the model fit indices, diagnostic criteria and class count (%) for the five models generated from the sample of 462 children. A 3-class model was accepted based on lowest Akaike information criterion, entropy=0.844, count in smallest class and significant Lo–Mendell–Rubin test over a 2-class model (p=0.0107). The class average probabilities across the subscales (figure 1C) portrays a clearer identification of an externalising symptom class (high probabilities of high-risk on both the *conduct* and *hyperactivity* subscales, n=37 or 8%), and another class presenting an internalising phenotype (higher probabilities in the *emotion* and *peer problems* subscales, n=19 or 4.1%). This 3-class model allowed for exploring relations to secondary anthropometric outcomes and eating behaviours with the remaining class defined as ‘Well-Adjusted’ (n=406 or 87.9%) and used as the reference for comparisons. Female sex was negatively associated with the externalising class membership (standardised beta coefficient (SE)=−1.74 (0.64), p=0.007) but not associated with the internalising class (beta=0.08 (0.59), p=0.894), as reflected in the probabilities of males in the externalising class=81.3%, internalising class=38.9% and 45.7% in the well-adjusted class.

In light of the above, 1–5 class LCA models were run for each sex separately (online supplemental figure S3, online supplemental tables S7 and S8). Although a similar 3-class model could be identified among boys, the conclusions were not clear for the girls and hence all further analyses of outcomes were performed on the LCA classes derived from the entire sample, adopting sex as a covariate where appropriate.

### Class specific anthropometry and eating behaviour outcomes

Figure 1D shows that children in the externalising profile were heavier for their age (difference: 0.46 (0.09 to 0.83) standardised units), reflected also in higher BMI for age (0.57 (0.25 to 0.88)) and larger waist circumference (0.49 (0.12 to 0.86)). In contrast, the children in the internalising class did not differ on these anthropometric outcomes compared with the well-adjusted children. Both internalising and externalising phenotypes presented higher food fussiness (0.58 (0.14 to 1.02) and 0.72 (0.21 to 1.23), respectively). The internalising class showed also less enjoyment of food (−0.63 (−1.21 to −0.047)) and higher level of desire to drink (0.78 (0.21 to 1.35)).

## Discussion

The rapid rise in the prevalence of obesity among women worldwide^1^ has been associated with a parallel increase in obesity among women in antenatal care. This study is one of the few to have addressed the impact of maternal obesity on offspring mental health in early childhood and highlights that increases in the risk of child obesity and psychopathology can be seen as early as 3–4 years. Considering influences of in utero exposures, our data suggest that maternal antenatal depression and placental function, rather than inflammation and glycaemic status, have a direct impact on these associations. Importantly, our final theoretically driven model explained 44.5% of the variance in the outcome.

In this sample, 11.3% of the UPBEAT children were high-risk of any psychopathology, twice higher than expected by British norms for preschoolers on the SDQ (ie, 5.3%, www.sdqinfo.org). Our sample also showed a larger divide than anticipated between sexes, one in five boys and 1 in 10 girls having high risks of hyperactivity or conduct problems, which at this age is predictive for developmental and clinical diagnoses of autism and ADHD at 5 years of age.^40^ Through the LCA approach, we showed that one in eight children born of women with obesity were likely to have some psychological/emotional issues (8% of children had externalising phenotypes and 4.1% internalising). We demonstrated that an externalising phenotype, rather than internalising psychological profile, was associated with a substantial increase in adiposity compared with well-adjusted children. This fills a gap in evidence on these two outcomes which are rarely combined and studied in such a young sample, but also mirrors the evidence to suggest that diagnosis of ADHD, combined type and autism are associated with increased adiposity in older children (ie, when diagnostic assessment is accessible) and adults.^14 51 52^ We suggest therefore an early potential predisposition to obesity at preschool age accompanies externalising symptoms, which in turn may form part of later diagnosis of these neurodevelopmental disorders.

Among the externalising group of children, we suggest the reported association may be mediated by increased food fussiness and possibly in food responsiveness (figure 1C). Food fussiness reflects a pattern of selective eating characterised by reluctance to try new foods, limited acceptability of a variety of foods and rejection of foods before tasting them. In contrast, food responsiveness reflects an increased tendency to eat in response to external food cues and food availability. Together, these behaviours may contribute to less favourable dietary patterns, the seeking of specific food types and consequently a greater adiposity among children exhibiting externalising symptoms. We hypothesise that child eating behaviours could develop in parallel to psychological traits via a neurological overlap whereby a child seeks high calorie/high sugar diet which in turn precipitates or enhances externalising behaviours. The developing central reward and hypothalamic tracts that control emotions, eating behaviour and the food response provide the most likely interacting neuronal networks to be affected by exposure to maternal obesity.^53^ Potential disruption in early childhood, or even antenatally, would predicate the need for early intervention.^54 55^

### Pathways to adverse child outcomes

If relationships between maternal obesity and suboptimal child outcomes have origins in early stages of neurodevelopment, identifying the strongest risk factors and the windows for early intervention requires a strong epidemiological framework. In this sample exclusively exposed to antenatal maternal obesity, depression and lower values of PlGF (placental function/angiogenic activity) have distinct direct relationships with psychological difficulties in the child. Thereby, we improve on previous prediction models which variably and inconsistently implicated obesity, GDM or inflammation in associations with offspring ADHD, autism or externalising/internalising outcomes, when neither have routinely been probed for maternal depression or candidate biological markers.^7 9 26 56–62^ Obesity is reported to be a state of mild (meta) inflammation, but the lack of a direct effect of the inflammatory cytokine IL-6 in the present study would argue against a role for mild activation in these associations. Furthermore, our findings concur with one previous study reporting no GDM relationship with child externalising/internalising symptoms once adjusted for maternal postnatal depression.^63^ We also showed that other statistically significant paths in our model reflected known biological associations (eg, maternal adiposity is positively associated with inflammation) which increases confidence in the validity of the models tested.

The lower value of maternal PlGF as observed in relation to risk of child psychopathology is indicative of reduced functional capacity of the placental syncytiotrophoblast, the epithelial cell layer across which nutrients in the maternal circulation and oxygen gain access to the fetal circulation. Low values of PlGF are associated with fetal growth restriction, pre-eclampsia and stillbirth.^64^ Poor nutrient supply to the fetus has been widely implicated in suboptimal neurodevelopment.^65^ In those women with obesity who develop placental dysfunction,^66^ this could play a role in the causal pathways leading to abnormal neurodevelopment. The effect size of −0.29 (−0.47 to −0.10) for the placental function is moderate but not negligible for a blood biomarker. In addition to the antenatal depression effect of 0.26 (0.06 to 0.46), these findings should incentivise further exploration and screening across the pregnant population. The biological mechanisms related to depression involve the effect of stimulation of the maternal hypothalamic–pituitary–adrenal pathway, and cortisol in particular, which is known to impact on placental and fetal brain growth.^67–69^ In mouse models, maternal stress is additionally associated with changes in the offspring gut microbiome in a sex-dependent manner,^70^ and of changes the paraventricular nucleus of the hypothalamus.^71^

While the novel models reported here emphasise parsimony and ease of interpretation, further steps towards a fuller understanding of the complex relationship between maternal depression and offspring neurodevelopment/adiposity are warranted. We recognise this requires disentangling other fetal and postnatal exposures linked to maternal antenatal depression as described previously in this sample^29^ and reported by others,^72 73^ particularly antenatal maternal infection, diet and changes in the fetal metabolome.

### Strengths and limitations

The main strength of this study lies in the implementation of SEM tools and the availability of repeated measures in a large sample to generate complex SEMs and to categorise children into latent profiles. Our latent constructs improved on previous literature relying on dichotomisation and single point measures such as depression, glycaemia, inflammation and placental function. This methodology offered superior control over measurement error and parameter calculations compared with conventional regression analyses and support testing theoretically driven associations. We also report good model fit for all models included and stable estimates encompassing previous effects known in the literature and observed in this cohort.^29^ Our results were not confounded by very early preterm births (<34 weeks) and pre-eclamptic pregnancies. These strengths combine to increase the likelihood that maternal antenatal depression and placental function drive the effects on child health over maternal obesity alone, glycaemic status or inflammation.

Some consideration should be given to the interpretation of the data. First, the study sample differed slightly in the demographic profile from the initial UPBEAT cohort and because of substantial attrition the sample size at follow-up was not large enough to perform comparisons by sex. We acknowledge this may limit the generalisability of our findings. It is possible that boys and girls differ in the onset and nature of externalising and internalising symptoms, which could associate with distinct anthropometric and eating behaviour outcomes. Fetal exposures may interact with offspring sex, for example, in a vulnerability in the male fetal brain immune response to a maternal stress in pregnancy.^74–76^ Similarly, while not tested here, it is possible that when maternal prenatal and postnatal depression is high, GDM affects these child symptoms in males only.^77^ Second, the directionality of the associations found between psychological profiles and anthropometry and eating behaviour cannot be completely clarified. Third, other factors may be at play such as shared genetic traits between mother and child and unmeasured familial confounding factors,^78 79^ diet quality postpartum^80^ and levels of physical and social activity. We adjusted for postnatal depression to litigate its effect on the SDQ reporting by the mother and its postnatal effect but we recommend that future studies include multi-informant assessments available at preschool age to improve robustness and objectiveness of similar outcome measures. Fourth, we cannot infer that the LCA profiles extracted here (ie, externalising and internalising phenotypes) had significant long-term psychological outcomes and a follow-up of these children is indicated to answer this. Lastly, our models are associative and cannot infer on causality in that they relied on observational data. The SEMs assumed linear relationships among latent and observed variables. It is possible that latent constructs did not fully capture their intended theoretical constructs. Further limitations include sensitivity to sample size in subgroup analyses on anthropometry and eating behaviour, and that measurement invariance across subgroups was not formally tested in all models.

### Clinical implication

We show that children exposed to maternal obesity have higher than expected adiposity by WHO standards at 3–4 years of age but also higher than expected elevated risk of both internalising and externalising psychological difficulties. We present evidence that children with an externalising phenotype had larger body size than those classified as ‘well-adjusted’. We clarify that the aetiology may be rooted in maternal depression and placental function, offering a specific mechanistic pathway that could be targeted through intervention. In evaluation of such an intervention and the issue of attrition and loss to follow-up among the children, routinely collected outcomes could be obtained from electronic health record. If such targeted interventions are empirically supported, they have the potential to alter health trajectories across two generations at the earliest timepoint possible. Such endeavour would require policies that address the dynamic interplay between socioeconomic conditions, lifestyle and access to care in order to support mental well-being of parents and future parents in all sections of society.

## Supplementary material

10.1136/bmjment-2026-302738 online supplemental file 1

## Data Availability

Data are available upon reasonable request.
