# Supplementary material for: Psychological and anthropometric outcomes in preschool children exposed to antenatal maternal obesity: modelling of early life risk factors
Source: BMJ Ment Health. 2026 Oct 12;29(1):e302738. doi: 10.1136/bmjment-2026-302738 (PMC13662093; doi:10.1136/bmjment-2026-302738)
Supplement: Supplementary file 1 [file bmjment-29-1-s001.pdf]

Supplemental File: Psychological and anthropometric outcomes in preschoolers exposed to antenatal maternal obesity: modelling of early life risk factors.

Sigurdardottir et al.,

Contents

Participants characteristics **2**

Child outcome measures, distributions and tables **6**

Model convergence, fit and selection criteria in *Mplus* **9**

SEM specification and estimation **9**

Path models 1 to 4 diagrams **10**

Latent Class Analyses (LCA), model fit and ouputs **11**

LCA by sex **11**

SEM outputs **13**

MPLUS OUTPUT MODEL 4 **15**

MPLUS OUTPUT MODEL 4 - adjusted for maternal latent SES **18**

References **22**

Participants characteristics

Table S1: Maternal factors between children who were included and excluded.

	Excluded	Included	p
n	1,024	462	
**Baseline**			
BMI (median [IQR])	35.40 [32.90, 38.92]	34.70 [32.60, 37.80]	0.004
Age(years) (mean (SD))	31.11 (5.51)	32.29 (5.29)	<0.001
Main ethnicity (%)			0.013
White	618 (60.4)	318 (68.8)	
Black	277 (27.1)	103 (22.3)	
Asian	68 (6.6)	19 (4.1)	
Other	61 (6.0)	22 (4.8)	
IMD (%)			0.077
Least Deprived	32 (3.1)	27 (5.9)	
2nd quintile	66 (6.5)	32 (7.0)	
3rd quintile	115 (11.3)	51 (11.1)	
4th quintile	344 (33.7)	165 (35.9)	
Most deprived	463 (45.4)	185 (40.2)	
Income (%)			<0.001
< £12,688	213 (20.8)	61 (13.2)	
£12,688 - £17,628	118 (11.5)	47 (10.2)	
£17,629 - £23,452	93 (9.1)	32 (6.9)	
£23,453 - £32,500	120 (11.7)	62 (13.4)	
> £32,500	310 (30.3)	208 (45.0)	
Prefers not to answer	170 (16.6)	52 (11.3)	
Highest Education (%)			0.011
None	49 (4.8)	10 (2.2)	
GCE (or equivalent)	178 (17.4)	66 (14.3)	
Vocational qualification	245 (23.9)	113 (24.5)	
A level (or equivalent)	173 (16.9)	66 (14.3)	
First degree	261 (25.5)	134 (29.0)	
Higher degree	118 (11.5)	73 (15.8)	
Born in the UK (%)	674 (65.8)	328 (71.0)	0.056
Nulliparous (%)	427 (41.7)	223 (48.3)	0.021
Smoking (%)			0.004
Never	653 (63.8)	335 (72.5)	
Current	82 (8.0)	19 (4.1)	
Ex - gave up before pregnancy	185 (18.1)	69 (14.9)	
Ex - gave up in pregnancy	104 (10.2)	39 (8.4)	
Intervention (%)	521 (50.9)	227 (49.1)	0.571
**Pregnancy outcomes**			
All GDM (%)	221 (26.4)	105 (24.1)	0.405
GDM only (%)	187 (22.2)	105 (24.1)	0.493
All PE (%)	89 (8.8)	0 (0.0)	<0.001
PE only (%)	50 (5.0)	0 (0.0)	<0.001
EPDS 1st visit (median [IQR])	6.00 [3.00, 10.00]	6.00 [3.00, 9.00]	0.464
EPDS 2nd visit (median [IQR])	5.00 [2.00, 9.00]	5.00 [3.00, 9.00]	0.481
EPDS 3rd visit (median [IQR])	5.00 [2.00, 8.00]	5.00 [2.00, 8.00]	0.813

Note:
In UPBEAT 1490 women had confirmed live infants and is the total sample size considered for the comparision against the 462 dyads who were included. Normally and non-normally distributed variables are compared with a t-test and a Kruskal-Wallis test, respectively. Categorical variables were analysed with a Chi-squared test. IMD: Index of Multiple Deprivation; BMI: Body Mass Index. EPDS: Edinburgh Postnatal Depression Scale, GDM: Gestational Diabetes Mellitus, PE:Pre-eclampsia.

Table S2: Birth factors between children who were included and excluded.

	Excluded	Included	p
n	1,024	462	
Gestational age at birth (days) (median [IQR])	278.00 [270.00, 286.00]	280.00 [272.00, 287.00]	0.013
Born <37 weeks (%)	73 (7.1)	15 (3.2)	0.005
Female (%)	495 (48.5)	237 (51.3)	0.343
Apgar (median [IQR])	10.00 [9.00, 10.00]	10.00 [9.00, 10.00]	0.599
WHO centile (median [IQR])	52.39 [29.46, 76.73]	60.06 [33.18, 79.39]	0.027
SGA WHO 10% (%)	57 (5.6)	29 (6.3)	0.682
LGA WHO 90% (%)	119 (11.7)	58 (12.6)	0.683
All C-section (%)	360 (35.3)	172 (37.2)	0.500
Emergency C-section (%)	160 (15.7)	87 (18.8)	0.155

Note:
Normally and non-normally distributed variables are compared with a t-test and a Kruskal-Wallis test, respectively. Categorical variables were analysed with a Chi-squared test. LGA: Large for gestational age, SGA: Small for gestational age.

Table S3: Data availability.

Variable	Valid	Missing
Child Sex	462(100.0%)	0(0.0%)
**SDQ**		
SDQ Total Score	462(100.0%)	0(0.0%)
Emotion total	462(100.0%)	0(0.0%)
Peer total	462(100.0%)	0(0.0%)
Hyperactive total	462(100.0%)	0(0.0%)
Conduct total	462(100.0%)	0(0.0%)
**CEBQ**		
Food responsiveness	459(99.4%)	3(0.6%)
Emotional over-eating	459(99.4%)	3(0.6%)
Emotional under-eating	459(99.4%)	3(0.6%)
Slowness in eating	459(99.4%)	3(0.6%)
Desire to drink	459(99.4%)	3(0.6%)
Food fussiness	459(99.4%)	3(0.6%)
Satiety responsiveness	459(99.4%)	3(0.6%)
Enjoyment of food	459(99.4%)	3(0.6%)
**Anthropometry**		
Height for age z-score	438(94.8%)	24(5.2%)
Weight for age z-score	440(95.2%)	22(4.8%)
Weight for height z-score	435(94.2%)	27(5.8%)
BMI for age z-score	435(94.2%)	27(5.8%)
Weight (kg)	441(95.5%)	21(4.5%)
Height (cm)	440(95.2%)	22(4.8%)
Waist (cm)	428(92.6%)	34(7.4%)
Sum of skinfold (mm)	348(75.3%)	114(24.7%)
Arm circumference	423(91.6%)	39(8.4%)
**Birth**		
Gestational age at birth (days)	462(100.0%)	0(0.0%)
Preterm birth <37 weeks	462(100.0%)	0(0.0%)
Apgar	455(98.5%)	7(1.5%)
Birthweight	462(100.0%)	0(0.0%)
SGA WHO 10%	462(100.0%)	0(0.0%)
LGA WHO 90%	462(100.0%)	0(0.0%)
All C-section	462(100.0%)	0(0.0%)
Emergency C-section	462(100.0%)	0(0.0%)
**Maternal factors**		
BMI	462(100.0%)	0(0.0%)
Age(years)	462(100.0%)	0(0.0%)
Main ethnicity	462(100.0%)	0(0.0%)
IMD	460(99.6%)	2(0.4%)
Income	462(100.0%)	0(0.0%)
Highest Education	462(100.0%)	0(0.0%)
Born in the UK	462(100.0%)	0(0.0%)
Smoking	462(100.0%)	0(0.0%)
Nulliparous	462(100.0%)	0(0.0%)
GDM	436(94.4%)	26(5.6%)
Intervention	462(100.0%)	0(0.0%)
EPDS 1st visit	383(82.9%)	79(17.1%)
EPDS 2nd visit	381(82.5%)	81(17.5%)
EPDS 3rd visit	371(80.3%)	91(19.7%)

Note:
IMD: Index of Multiple Deprivation; BMI: Body Mass Index. EPDS: Edinburgh Postnatal Depression Scale, GDM: Gestational Diabetes Mellitus, LGA: Large for gestational age, PE:Pre-eclampsia, SGA: Small for gestational age.

Waist was measured at the halfway point between the inferior margin of the lowest rib and the iliac crest. Mid arm circumference was measured from the tip of the elbow to the edge of the acromion. Skinfold thicknesses were measured using Harpenden skinfold callipers. Sum of skinfolds thicknesses was calculated as the sum of abdominal, subscapular, suprailiac, triceps and biceps skinfolds.

Child outcome measures, distributions and tables

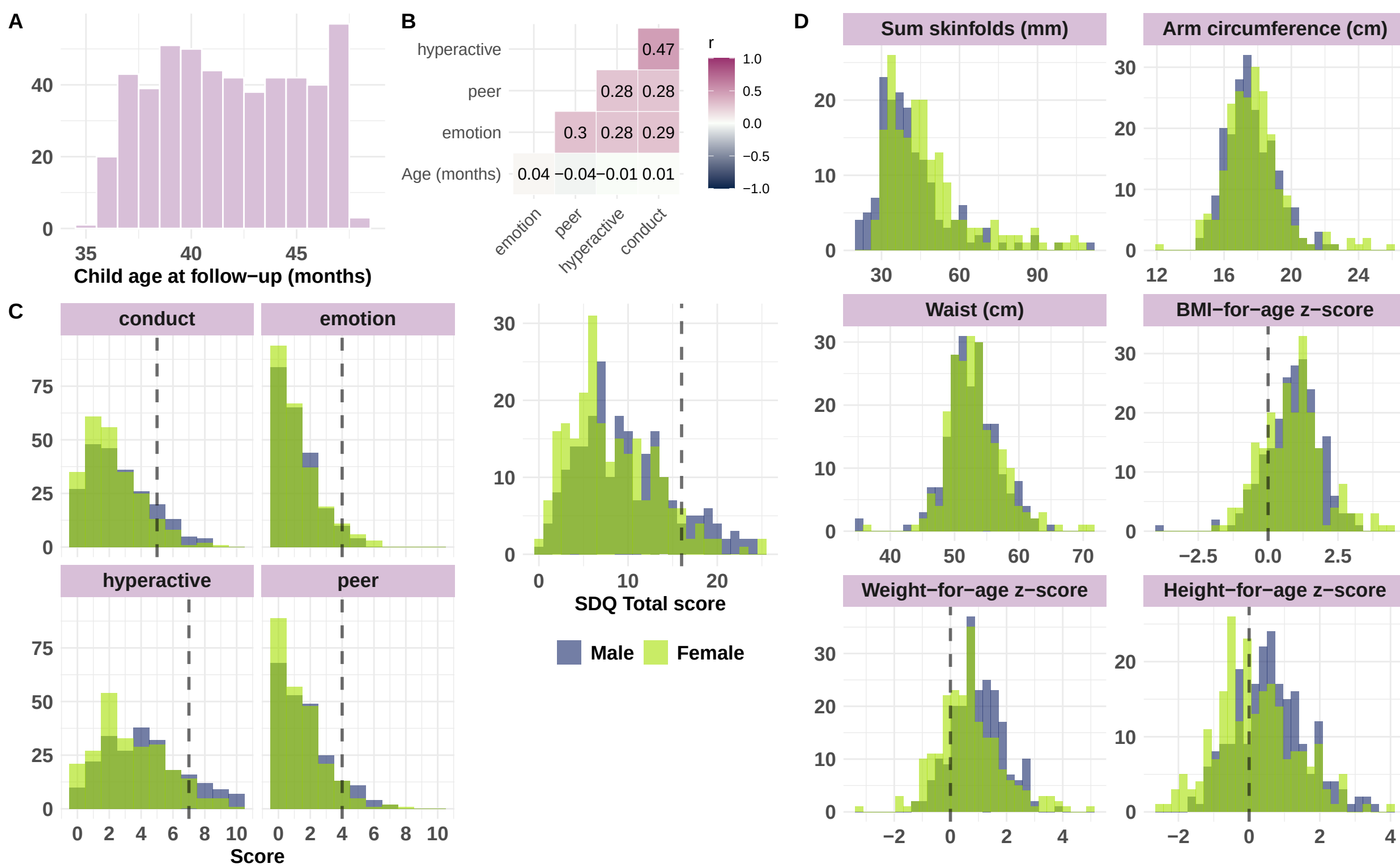

Figure S1: A: Age distribution of n=462 children at follow-up visit. B:Correlation matrix of the SDQ subscales and child age (Spearman r). C: Distribution of SDQ total and subscales scores. Vertical lines represent the standard cut-offs for normal/low vs high/very high risk. D: Distributions of anthropometric measurements. E: Latent Class Analysis on the subscales the Strength and Difficulty Questionnaire [SDQ]. Y-axis: average class probability of being at high-risk (vs low-risk) on each subscale for each n-class model. A 3-class model was accepted based on assessment of model fit and diagnostic criteria. F: Externalising (n=37 [8%]) and internalising classes (n=19 [4.1%]) were compared against the well-adjusted class (n=406 [87.9%]) on anthropometric and eating behaviour outcomes where each model included child sex as a covariate when appropriate.

Table S4: Birth outcomes by sex.

	Overall	Male	Female	p
n	462	225	237	
Gestational age at birth (days) (median [IQR])	280.00 [272.00, 287.00]	280.00 [272.00, 288.00]	280.00 [272.00, 287.00]	0.530
Preterm birth <37 weeks (%)	15 (3.2)	11 (4.9)	4 (1.7)	0.093
Apgar (median [IQR])	10.00 [9.00, 10.00]	10.00 [9.00, 10.00]	10.00 [9.00, 10.00]	0.280
Birthweight (mean (SD))	3,511.18 (490.45)	3,560.77 (506.58)	3,464.09 (470.87)	0.034
SGA WHO 10% (%)	29 (6.3)	12 (5.3)	17 (7.2)	0.533
LGA WHO 90% (%)	58 (12.6)	27 (12.0)	31 (13.1)	0.834
All C-section (%)	172 (37.2)	95 (42.2)	77 (32.5)	0.039
Emergency C-section (%)	87 (18.8)	49 (21.8)	38 (16.0)	0.144

Note:
462 dyads were included. Normally and non-normally distributed variables are compared with a t-test and a Kruskal-Wallis test, respectively. Categorical variables were analysed with a Chi-squared test. SGA: Small for gestational age, LGA: Large for gestational age. WHO: World Health Organisation. WHO centiles are adjusted for sex and gestational age at delivery.

Table S5: Anthropometry, eating behaviour and birth outcomes by High/Low SDQ total risk

	Boys			Girls		
	Low-risk	High-risk	p	Low-risk	High-risk	p
n	192	33		218	19	
Age (months) (mean (SD))	41.87 (3.35)	42.03 (3.52)	0.801	41.74 (3.45)	40.11 (2.98)	0.047
SDQ Total Score (median [IQR])	8.00 [5.00, 11.00]	19.00 [17.00, 21.00]	<0.001	6.50 [4.00, 10.00]	18.00 [16.00, 19.50]	<0.001
**Anthropometry**						
BMI for age z-score (mean (SD))	0.84 (0.98)	1.27 (0.80)	0.024	0.85 (1.13)	0.87 (0.95)	0.941
Height for age z-score (mean (SD))	0.58 (0.98)	0.85 (1.26)	0.172	0.17 (1.13)	0.21 (1.19)	0.894
Weight for age z-score (mean (SD))	0.93 (0.87)	1.34 (0.98)	0.017	0.68 (1.18)	0.71 (1.12)	0.894
Waist (cm) (mean (SD))	52.43 (4.06)	54.81 (4.05)	0.004	53.29 (4.27)	51.91 (4.89)	0.195
Sum of skinfold (mm) (median [IQR])	38.15 [32.18, 46.23]	41.40 [37.12, 49.20]	0.196	44.20 [35.40, 53.30]	39.70 [33.80, 51.70]	0.512
Arm circumference (mean (SD))	17.54 (1.45)	18.19 (1.61)	0.031	17.84 (1.89)	17.42 (1.43)	0.364
**Eating behaviour**						
Food responsiveness (median [IQR])	2.00 [1.40, 2.60]	2.20 [1.50, 3.00]	0.219	2.00 [1.40, 2.60]	2.20 [1.70, 2.60]	0.420
Satiety responsiveness (mean (SD))	3.10 (0.70)	2.93 (0.76)	0.209	3.14 (0.77)	3.21 (1.05)	0.700
Emotional under-eating (mean (SD))	2.70 (0.99)	2.88 (1.03)	0.343	2.54 (0.89)	3.12 (1.02)	0.008
Emotional over-eating (median [IQR])	1.25 [1.00, 1.75]	1.50 [1.00, 1.75]	0.586	1.25 [1.00, 1.50]	1.00 [1.00, 1.75]	0.923
Food fussiness (mean (SD))	2.90 (0.91)	3.46 (0.90)	0.002	2.81 (0.94)	3.30 (1.20)	0.035
Slowness in eating (mean (SD))	3.09 (0.84)	3.28 (0.91)	0.240	3.13 (0.86)	3.32 (1.09)	0.392
Enjoyment of food (mean (SD))	3.54 (0.91)	3.46 (0.98)	0.666	3.63 (0.87)	3.00 (1.07)	0.003
Desire to drink (median [IQR])	2.67 [1.67, 3.67]	3.33 [1.83, 4.00]	0.203	2.67 [1.67, 3.33]	3.33 [2.50, 4.67]	0.012
**Birth outcomes**						
Gestational age at birth (days) (median [IQR])	280.50 [272.00, 288.00]	278.00 [274.00, 284.00]	0.454	280.00 [273.00, 287.00]	274.00 [269.50, 286.00]	0.218
Preterm birth <37 weeks (%)	9 (4.7)	2 (6.1)	1.000	4 (1.8)	0 (0.0)	1.000
Apgar (median [IQR])	10.00 [9.00, 10.00]	10.00 [9.00, 10.00]	0.460	10.00 [9.00, 10.00]	10.00 [9.00, 10.00]	0.488
Birthweight (g) (mean (SD))	3,558.97 (499.56)	3,571.27 (553.75)	0.898	3,473.08 (467.58)	3,360.95 (509.00)	0.321
SGA WHO 10% (%)	11 (5.7)	1 (3.0)	0.827	14 (6.4)	3 (15.8)	0.292
LGA WHO 90% (%)	22 (11.5)	5 (15.2)	0.754	29 (13.3)	2 (10.5)	1.000
All C-section (%)	78 (40.6)	17 (51.5)	0.327	69 (31.7)	8 (42.1)	0.498
Emergency C-section (%)	39 (20.3)	10 (30.3)	0.291	35 (16.1)	3 (15.8)	1.000

Note:

BMI: Body mass index; LGA: Large for gestational age; SGA:Small for gestational age.

Model convergence, fit and selection criteria in *Mplus*

Model convergence was assessed from the Proportional Scale Reduction (PSR) factor [1], which should to be close to 1 (or <1.1 according to [2]) for the iterations after the burn-in. The residual variance is visualised for overlapp on a trace plots. Instead of the PSR criterion we used the limited *FBiteration* option rather than the bconvergence= 0.05 option to reduce computational time and so the PSR was checked manually. No thinning was performed as per recommendation [3]. ***Model fit*** was assessed by the Posterior predictive checking (PPC) and i.e. the Chi^2 test 95%CI exclude 0 and the Bayesian posterior predictive p-value is approximately 0.05 [4]. We report the median of the posterior distribution as the point estimate and the 5% Bayesian *credibility* interval (CI) [4]. Additionally, the posterior distribution histograms should also have smooth shape with no gaps and one peak and that the autocorrelation between lags (i.e. between iterations for a parameter) is not large.

Model fit indices and other diagnostic criteria were assessed to decide on which model to accept as follows: Bayesian information criterion (BIC), the Akaike information criterion (AIC,[5]), sample-size adjusted Bayesian information criterion (aBIC), where lower values infer better model fit [6]. The Vuong-Lo-Mendell-Rubin adjusted likelihood ratio test (LMR) indicates that if p<0.05 that an n-class model provides better fit than a n-1 class model [7]. Entropy indicates the degree of separation of individuals into classes [8] with a value over 0.60 deemed acceptable [9]. Model selection is also at the discretion of the researcher to interpret, basing their judgement on class count(%) and interpretability in light of theory [10].

SEM specification and estimation

To make it comparable to ML output, the Bayes estimation here included no priors. Bayes does not require large samples nor that the parameter estimates be normally distributed, and provides the credibility intervals (akin confidence intervals in frequentist statistics) which can be asymetrical [1]. Similar to the ML estimation, the Bayes estimation handles missing data under the missing-at-random missing data theory [1,11] to use all available data. In line with reporting guidelines [4,12], the following *Mplus* settings were used: 8 Markov chain Monte Carlo (MCMC) chains, GIBBS algorithm, FBIterations = 100000 to 200000 and processors=8. Latent factor indicators were free and the variance of the latent factors was fixed at 1.

Path models 1 to 4 diagrams

Model 1

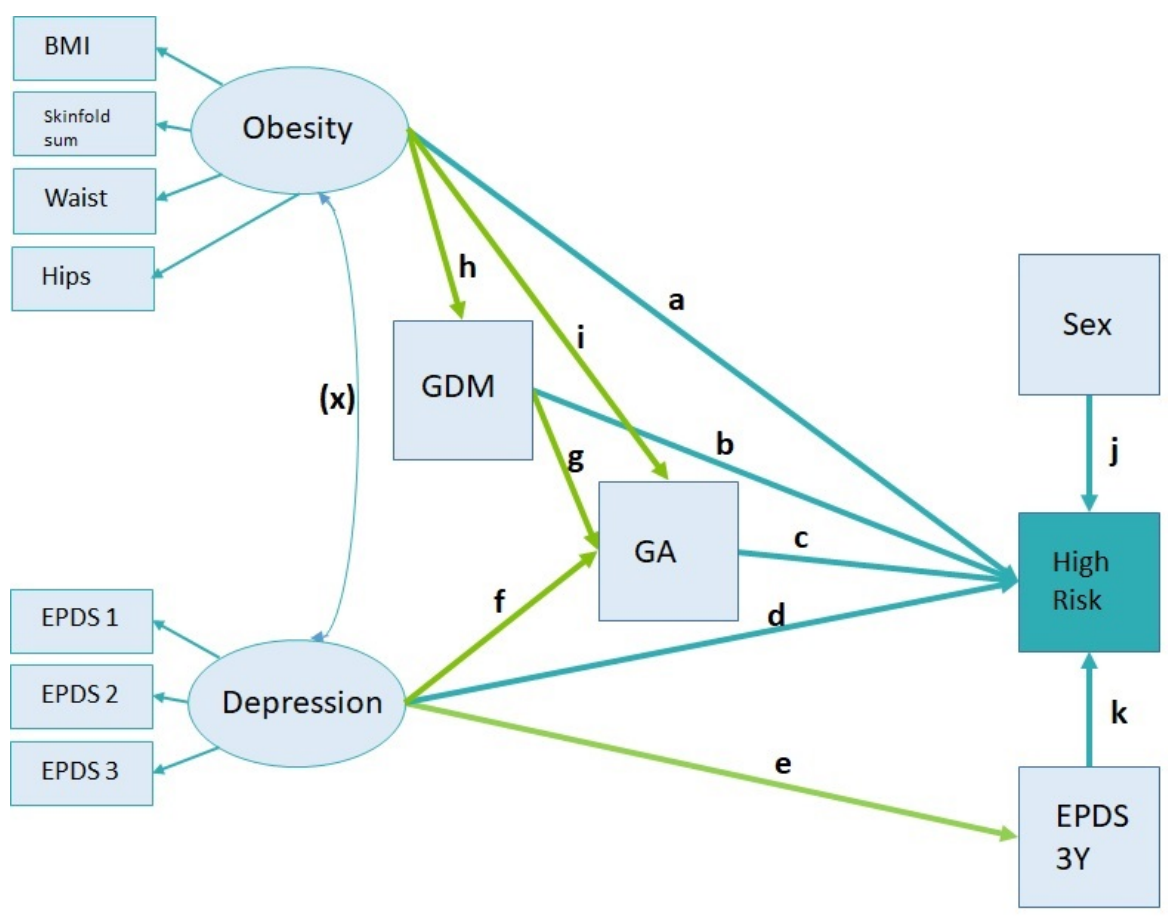

Model 2

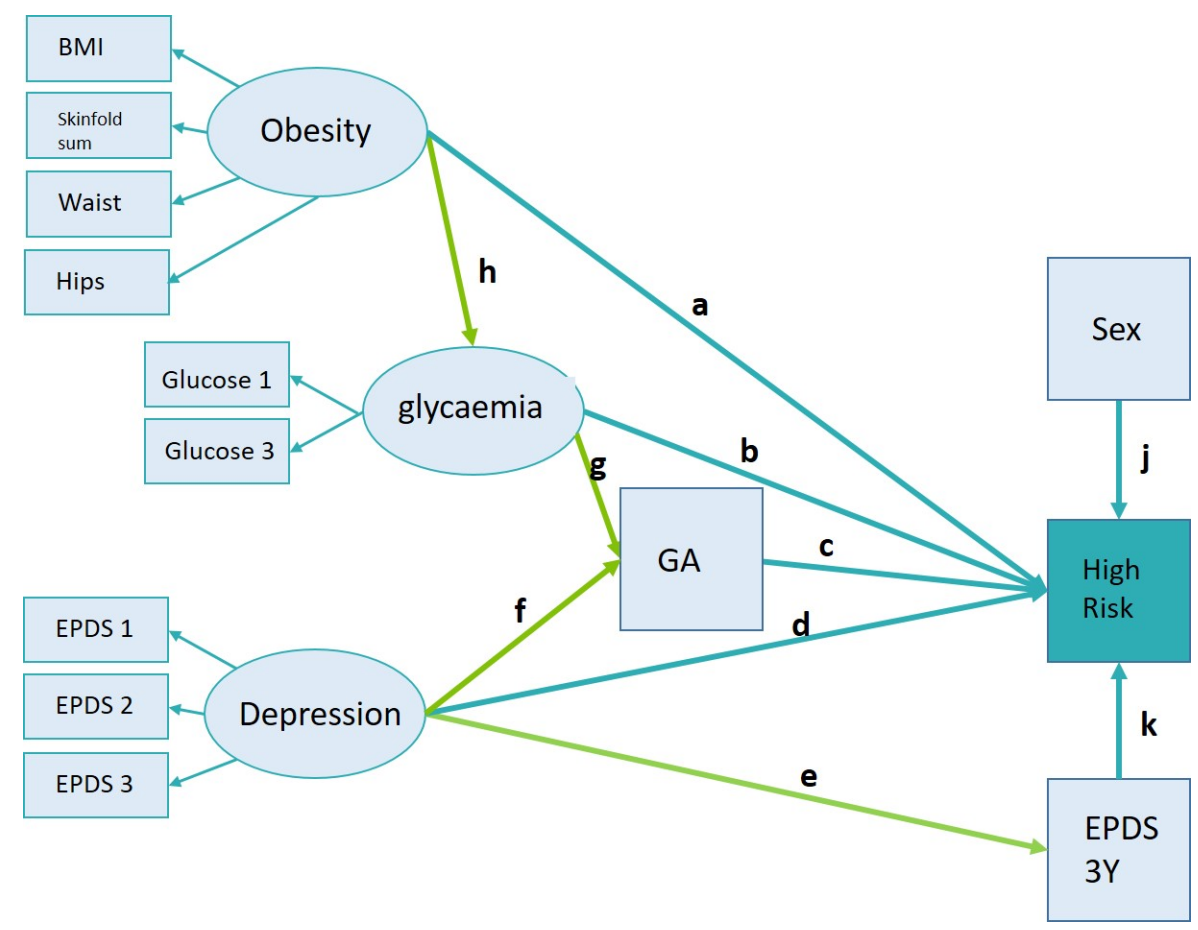

Model 3

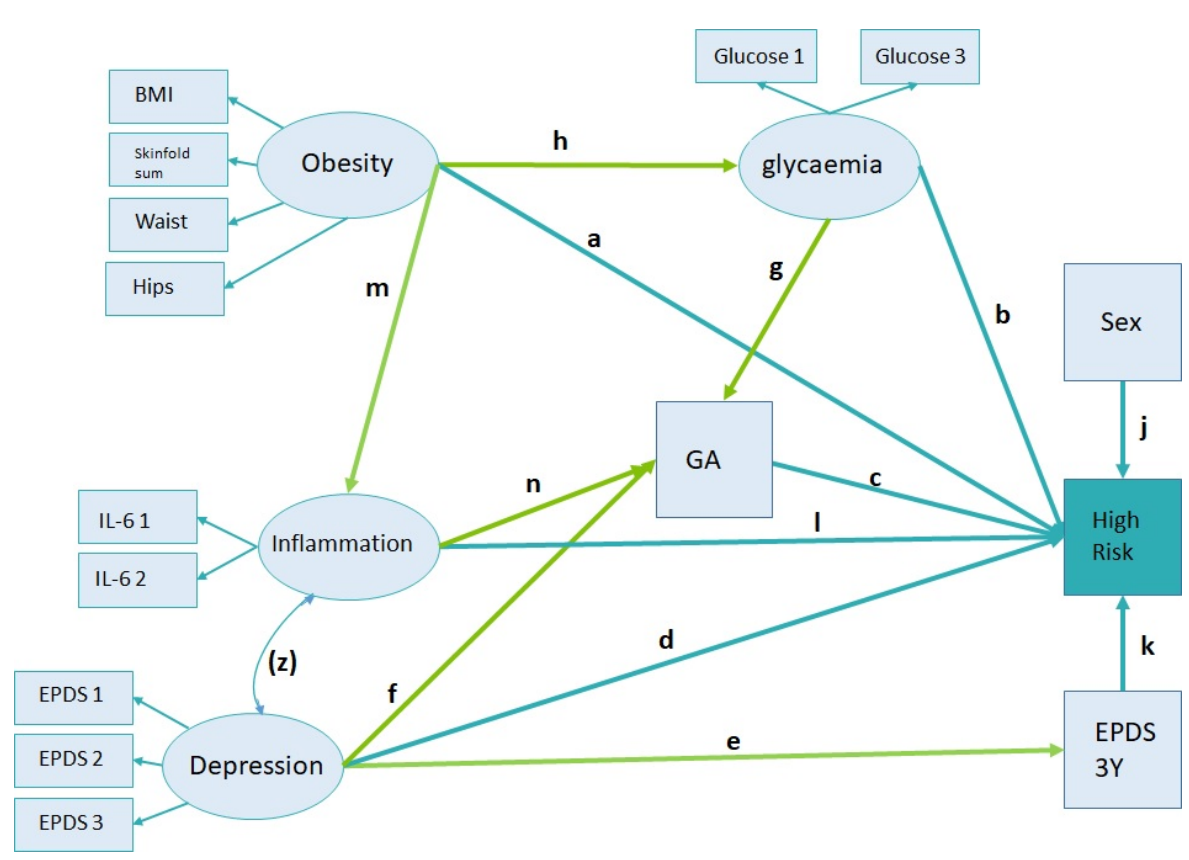

Model 4

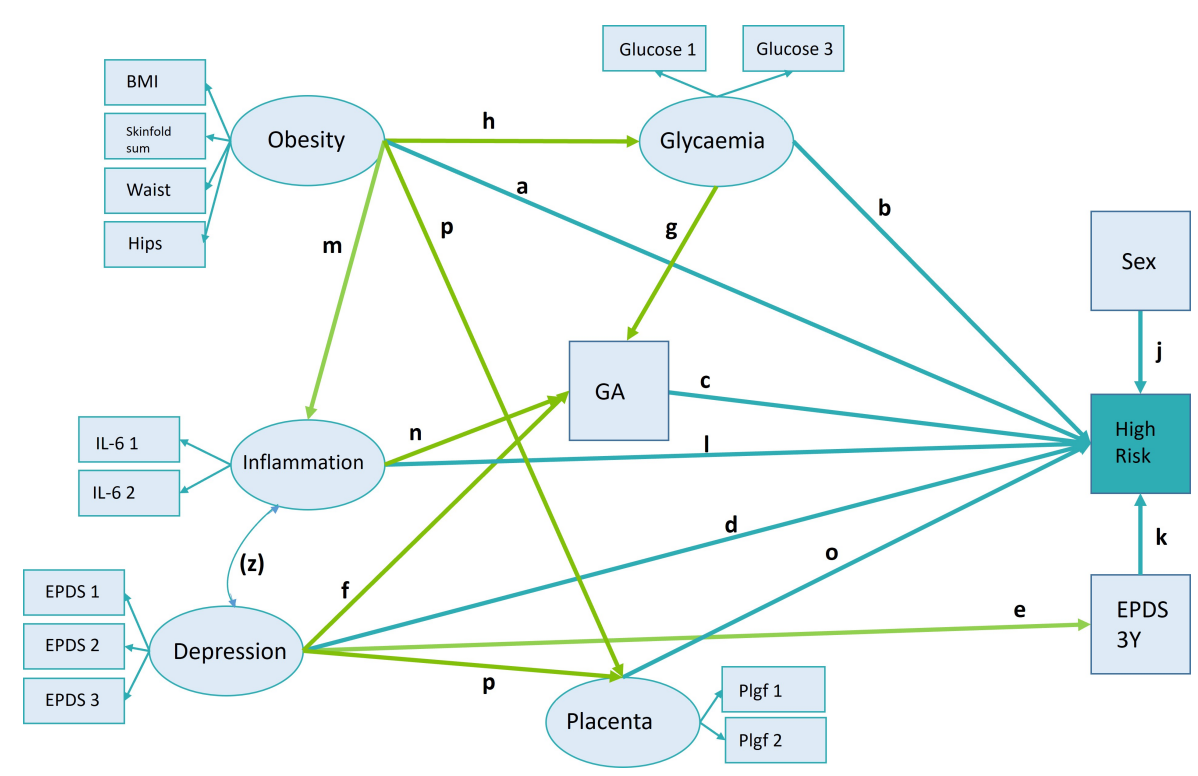

Figure S2: Structural equation models were specified and run from Model 1 and in increasing complexity to Model 4. Main direct effect paths of interest are in blue and indirect in green. Latent variables are depicted in ovals and observed variables in squares. Curved bidirectional paths refers to a correlation. Variances of the latent variables were fixed at 1. Residuals are not shown. Available data: Body Mass Index (BMI)=1357, sum of skinfold(SSF)=1338, Hip circumference=1346, Waist=1346, Edinburgh Postnatal Depression Scale 1 (EPDS 1)=1181, EPDS 2=1056, EPDS 3=932, gestational age (GA)=1357, gestational diabetes (GDM)=270 diagnosed and 887 not diagnosed, Glucose 1= 948, Glucose 3=735, High SDQ= 52 yes and 410 no, Interleukin-6 1 (IL-6 1)=905, IL-6 2=825, child sex=1357.

Latent Class Analyses (LCA), model fit and ouputs

Table S6: Latent Class Analysis in for 1 to 5 class models for total sample (n=462)

	Model fit and diagnostic criteria								Count(%) per class				
N-class Model	LL	Parameters	AIC	BIC	aBIC	Entropy	LMR	LMR p-value	Class 1	Class 2	Class 3	Class 4	Class 5
1	-670	4	1348	1365	1352				462(100%)				
2	-630	9	1277	1315	1286	0.66	78.12	0.00	80(17.3%)	382(82.7%)			
**3**	**-623**	**14**	**1273**	**1331**	**1287**	**0.84**	**13.55**	**0.01**	**37(8%)**	**406(87.9%)**	**19(4.1%)**		
4	-622	19	1282	1361	1300	0.93	1.13	0.44	2(0.4%)	34(7.4%)	411(89%)	15(3.2%)	
5	-622	24	1292	1391	1315	0.93	0.09	0.79	9(1.9%)	30(6.5%)	3(0.6%)	407(88.1%)	13(2.8%)

Note:
In bold is the 3-class model which was chosen based on predefined criteria. Entropy is the measure of class classification accuracy. AIC: Akaike Information Criterion, BIC: Bayesian Information Criterion, aBIC: Adjusted Bayesian Information Criterion, LL: Log-Likelihood, LMR: Lo–Mendell–Rubin Likelihood Ratio Test.

LCA by sex

Using the same assessment as previously, group likely count and proportion were: class 1= 14(6.2%) with an internalising phenotype, class 2= 23(10.2%) with an externalising phenotype and class 3=188(83.6%) showed low-risk on all subscales.

However, the 1-5 class models generated for the 237 girls did not yield as clear conclusions. The model fit indices present the lowest BIC for a 1-class model but lowest AIC and aBIC for a 2-class model. The LMR p-value is not significant at p=0.08 for the 2-class model nor the 3-class model (p=0.09). The likely class counts in the smallest class is 29(12.2%) in the 2-class model and 7(3%) in the 3-class and 4-class models and 5(2.1%) in the 5-class model.

Table S7: Latent Class Analysis in for 1 to 5 class models for girl (n=237)

	Model fit and diagnostic criteria								Count(%) per class				
N-class Model	LL	Parameters	AIC	BIC	aBIC	Entropy	LMR	LMR p-value	Class 1	Class 2	Class 3	Class 4	Class 5
1	-302	4	611	625	612				237(100%)				
**2**	**-288**	**9**	**595**	**626**	**598**	**0.73**	**25.18**	**0.08**	**29(12.2%)**	**208(87.8%)**			
3	-285	14	599	647	603	0.89	6.06	0.09	219(92.4%)	11(4.6%)	7(3%)		
4	-284	19	606	672	612	0.62	2.18	0.13	24(10.1%)	7(3%)	197(83.1%)	9(3.8%)	
5	-284	24	616	699	623	0.94	0.71	0.61	5(2.1%)	8(3.4%)	201(84.8%)	6(2.5%)	17(7.2%)

Table S8: Latent Class Analysis in for 1 to 5 class models in boys (n=225)

	Model fit and diagnostic criteria								Count(%) per class				
N-class Model	LL	Parameters	AIC	BIC	aBIC	Entropy	LMR	LMR p-value	Class 1	Class 2	Class 3	Class 4	Class 5
1	-360	4	729	742	730				225(100%)				
2	-331	9	680	711	682	0.64	56.27	0.00	159(70.7%)	66(29.3%)			
**3**	**-326**	**14**	**680**	**728**	**683**	**0.89**	**10.05**	**0.04**	**14(6.2%)**	**23(10.2%)**	**188(83.6%)**		
4	-325	19	688	753	693	0.90	1.43	0.41	6(2.7%)	23(10.2%)	185(82.2%)	11(4.9%)	
5	-325	24	698	780	704	0.75	0.22	0.65	3(1.3%)	8(3.6%)	6(2.7%)	185(82.2%)	23(10.2%)

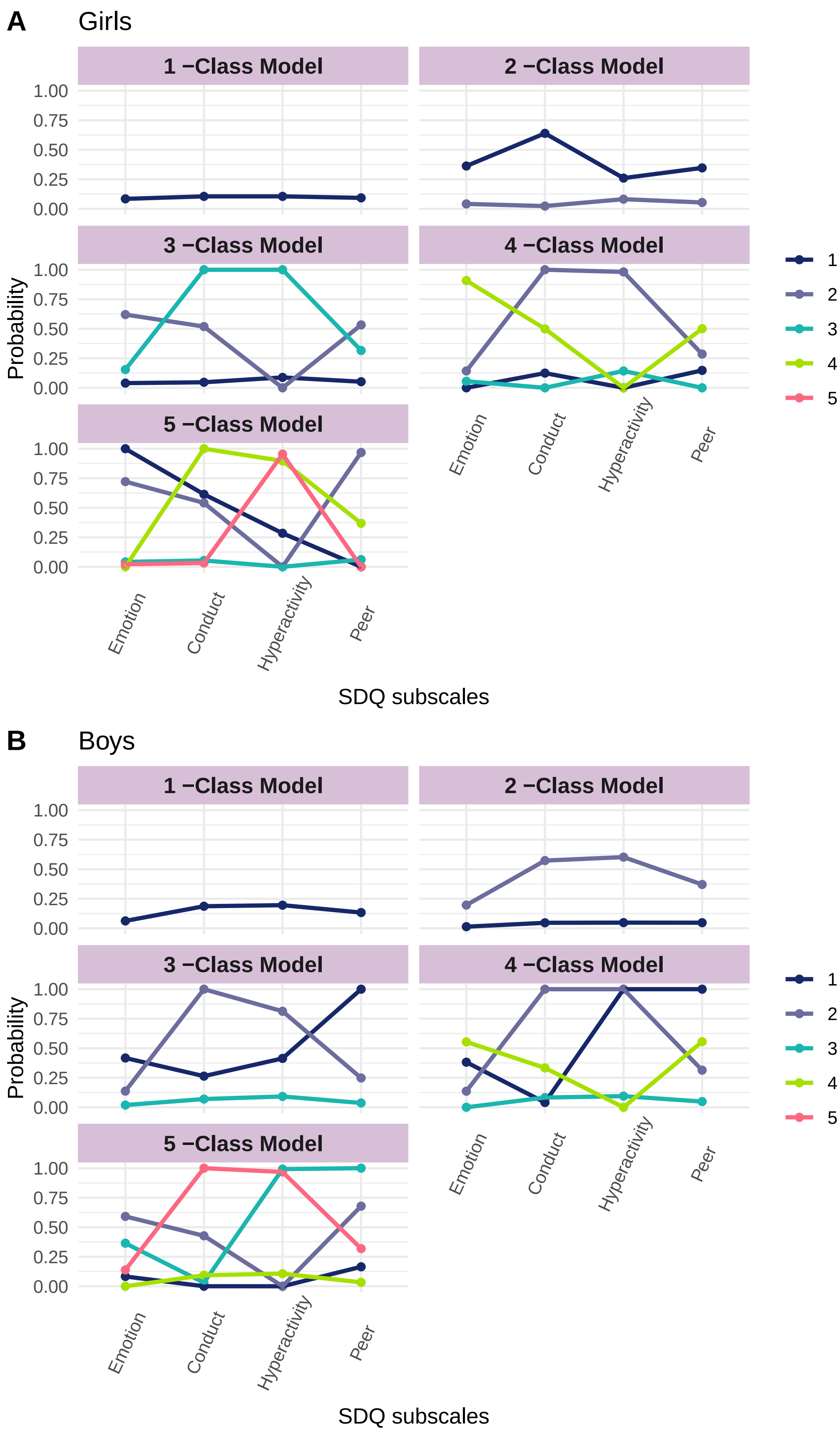

Figure S3: LCA was performed for girls and boys separately and class subscale probabilities plotted. A 3-class model was accepted for boys and a 2-class model of girls.

SEM outputs

Table S9: Model convergence and fit.

Model	Changes	Maximum PSR after burn-in	Chi-sq 95% CI	PP p-value	R^2 (95%CI) for high-risk
1		1.129	-8.58 to 64.4	0.06	0.16(0.07 to 0.28)
2	remove x and i paths, replace GDM with latent glycemia, FB iteration 200k	<1.1	-16.67 to 59.48	0.13	0.26(0.1 to 0.49)
3	add inflammation and paths m n z, FB iteration 200k	<1.1	-12.53 to 76.77	0.08	0.29(0.12 to 0.6)
4	add placenta and paths o, p, q, FB iteration to 300k	<1.1	-4.35 to 95.57	0.04	0.35(0.06 to 0.96)

Note:
PP p-value: posterior predicted p-value, PSR: Potential Scale Reduction. The Chi-squared test represent the 95% CI for difference between observed and replicated chi-square values.

The trace plots were not all visualised for practicality reasons but we refer to Figure S4 for examples.

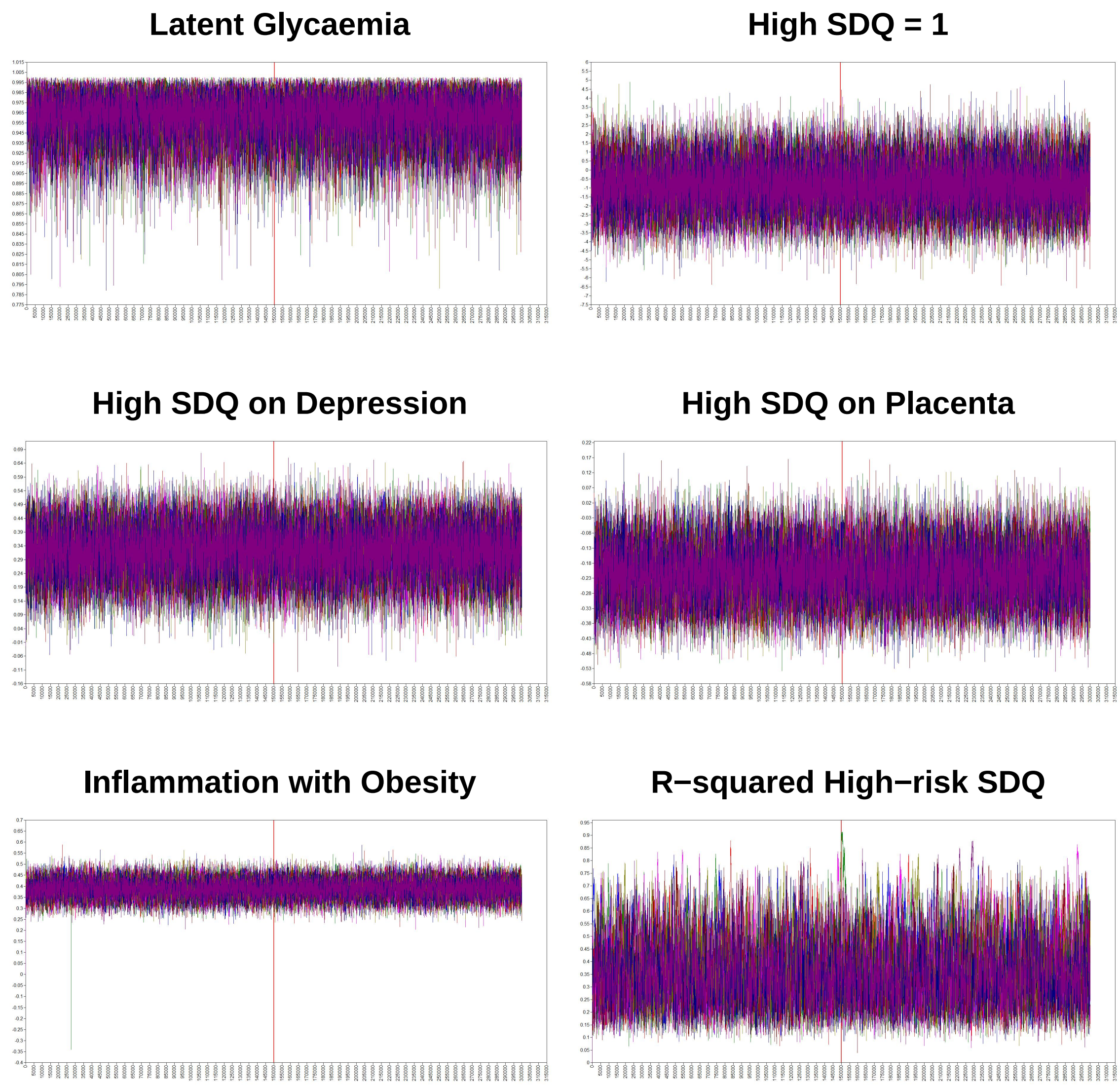

Figure S4: Example of trace plots derived from Model 3.

MPLUS OUTPUT MODEL 4

The measurement models for latent factors was good with standardized factor loadings of the indicators between 0.367 and 0.981 (p<0.001). See MPLUS output for MODEL 4 below.

```
STDYX Standardization

                               Posterior  One-Tailed         95% C.I.
                    Estimate       S.D.      P-Value   Lower 2.5%  Upper 2.5%  Significance
 DEPRESSI BY
    F1_EPDSTO          0.713       0.019      0.000       0.675       0.748      *
    F2_EPDSTO          0.896       0.014      0.000       0.868       0.923      *
    F3_EPDSTO          0.820       0.016      0.000       0.786       0.850      *

 OBESITY  BY
    BMI                0.981       0.012      0.000       0.967       0.994      *
    SF_SU              0.538       0.021      0.000       0.498       0.577      *
    WAIST              0.712       0.016      0.000       0.682       0.739      *
    HIP_CI             0.834       0.013      0.000       0.814       0.853      *

 GLYCEMIA BY
    B1_GLUCOS          0.367       0.077      0.000       0.227       0.527      *
    B3_GLUCOS          0.479       0.100      0.000       0.309       0.706      *

 PLACENTA BY
    B1_PLGF_L          0.866       0.091      0.000       0.659       0.991      *
    B2_PLGF_L          0.680       0.082      0.000       0.577       0.892      *

 INFLAM2  BY
    B1_IL6_LO          0.632       0.058      0.000       0.523       0.750      *
    B2_IL6_LO          0.568       0.053      0.000       0.466       0.673      *

 GLYCEMIA   ON
    OBESITY            0.186       0.060      0.001       0.069       0.306      *

 INFLAM2    ON
    OBESITY            0.390       0.044      0.000       0.304       0.474      *

 PLACENTA   ON
    DEPRESSION        -0.005       0.043      0.454      -0.091       0.077
    INFLAM2            0.001       0.058      0.493      -0.115       0.114
    OBESITY           -0.060       0.045      0.088      -0.151       0.027
    GLYCEMIA           0.108       0.070      0.057      -0.025       0.247

 PLACENTA   ON
    F7_NEO_SE         -0.001       0.036      0.487      -0.072       0.068

 GA_DEL ON
    DEPRESSION        -0.084       0.031      0.004      -0.144      -0.023      *
    GLYCEMIA          -0.230       0.063      0.000      -0.357      -0.107      *
    INFLAM2           -0.035       0.044      0.211      -0.121       0.051
    OBESITY            0.000       0.000      1.000       0.000       0.000
```

```
TOT3YEPDS  ON
   DEPRESSION         0.586        0.036        0.000         0.511        0.652      *

SDQHIGH    ON
   GLYCEMIA          -0.333        0.187        0.042        -0.687        0.045
   DEPRESSION         0.324        0.093        0.001         0.135        0.497      *
   OBESITY           -0.002        0.105        0.492        -0.210        0.200
   INFLAM2            0.056        0.135        0.340        -0.216        0.311
   PLACENTA          -0.216        0.090        0.011        -0.385       -0.033      *

SDQHIGH    ON
   GA_DEL             -0.063        0.049        0.101        -0.157        0.034
   TOT3YEPDS          0.003        0.087        0.487        -0.169        0.172
   F7_NEO_SE         -0.116        0.073        0.058        -0.256        0.029

INFLAM2  WITH
   DEPRESSION         0.070        0.054        0.098        -0.036        0.175
   GLYCEMIA           0.000        0.000        1.000         0.000        0.000

GLYCEMIA WITH
   DEPRESSION         0.000        0.000        1.000         0.000        0.000

OBESITY  WITH
   DEPRESSION         0.000        0.000        1.000         0.000        0.000

Intercepts
   F1_EPDSTO          1.452        0.041        0.000         1.372        1.533      *
   F2_EPDSTO          1.280        0.040        0.000         1.201        1.359      *
   F3_EPDSTO          1.240        0.041        0.000         1.159        1.322      *
   GA_DEL             27.446        0.530        0.000        26.413       28.492      *
   B1_GLUCOS          5.955        0.141        0.000         5.681        6.233      *
   B3_GLUCOS          4.536        0.124        0.000         4.295        4.781      *
   B1_IL6_LO          1.751        0.053        0.000         1.648        1.855      *
   B2_IL6_LO          1.246        0.046        0.000         1.156        1.336      *
   BMI                7.623        0.149        0.000         7.334        7.918      *
   SF_SU              4.475        0.091        0.000         4.297        4.654      *
   WAIST             10.138        0.198        0.000         9.753       10.529      *
   HIP_CI            11.928        0.232        0.000        11.476       12.386      *
   B1_PLGF_L          6.802        0.182        0.000         6.443        7.159      *
   B2_PLGF_L          8.276        0.212        0.000         7.861        8.693      *
   TOT3YEPDS          1.260        0.059        0.000         1.146        1.376      *

Thresholds
   SDQHIGH$1         -0.851        1.345        0.263        -3.449        1.832

Variances
   DEPRESSION         1.000        0.000        0.000         1.000        1.000
   OBESITY            1.000        0.000        0.000         1.000        1.000

Residual Variances
   F1_EPDSTO          0.491        0.027        0.000         0.440        0.544      *
   F2_EPDSTO          0.196        0.025        0.000         0.148        0.246      *
   F3_EPDSTO          0.328        0.026        0.000         0.278        0.382      *
   GA_DE              0.935        0.030        0.000         0.862        0.977      *
```

B1_GLUCOS	0.865	0.059	0.000	0.722	0.948	*
B3_GLUCOS	0.771	0.104	0.000	0.502	0.905	*
B1_IL6_LO	0.601	0.074	0.000	0.437	0.727	*
B2_IL6_LO	0.677	0.060	0.000	0.547	0.783	*
BMI	0.038	0.014	0.000	0.011	0.066	*
SF_SU	0.710	0.022	0.000	0.667	0.752	*
WAIST	0.493	0.021	0.000	0.453	0.535	*
HIP_CI	0.304	0.017	0.000	0.272	0.338	*
B1_PLGF_L	0.249	0.152	0.000	0.018	0.565	*
B2_PLGF_L	0.538	0.120	0.000	0.204	0.667	*
TOT3YEPDS	0.657	0.042	0.000	0.575	0.739	*
GLYCEMIA	0.966	0.023	0.000	0.907	0.995	*
PLACENTA	0.979	0.018	0.000	0.930	0.996	*
INFLAM2	0.848	0.034	0.000	0.775	0.908	*

R-SQUARE

Variable	Estimate	Posterior S.D.	One-Tailed P-Value	95% C.I. Lower 2.5%	Upper 2.5%
SDQHIGH	0.346	0.131	0.000	0.163	0.675
F1_EPDSTOTAL	0.509	0.027	0.000	0.456	0.560
F2_EPDSTOTAL	0.804	0.025	0.000	0.754	0.852
F3_EPDSTOTAL	0.672	0.026	0.000	0.618	0.722
GA_DEL_DAY	0.065	0.030	0.000	0.023	0.138
B1_GLUCOSE	0.135	0.059	0.000	0.052	0.278
B3_GLUCOSE	0.229	0.104	0.000	0.095	0.498
B1_IL6_LOG2	0.399	0.074	0.000	0.273	0.563
B2_IL6_LOG2	0.323	0.060	0.000	0.217	0.453
BMI	0.962	0.014	0.000	0.934	0.989
SF_SUM	0.290	0.022	0.000	0.248	0.333
WAIST	0.507	0.021	0.000	0.465	0.547
HIP_CIRCUM	0.696	0.017	0.000	0.662	0.728
B1_PLGF_LOG2	0.751	0.152	0.000	0.435	0.982
B2_PLGF_LOG2	0.462	0.120	0.000	0.333	0.796
TOT3YEPDS	0.343	0.042	0.000	0.261	0.425

MPLUS OUTPUT MODEL 4 - adjusted for maternal latent SES

STANDARDIZED MODEL RESULTS

STDYX Standardization

	Estimate	Posterior S.D.	One-Tailed P-Value	95% C.I. Lower 2.5%	Upper 2.5%	Significance
DEPRESSI BY						
F1_EPDSTO	0.716	0.019	0.000	0.677	0.750	*
F2_EPDSTO	0.895	0.014	0.000	0.866	0.921	*
F3_EPDSTO	0.821	0.016	0.000	0.788	0.850	*
OBESITY BY						
BMI	0.982	0.023	0.000	0.968	0.996	*
SF_SU	0.538	0.024	0.000	0.497	0.576	*
WAIST	0.711	0.022	0.000	0.681	0.739	*
HIP_CI	0.833	0.021	0.000	0.812	0.852	*
GLYCEMIA BY						
B1_GLUCOS	0.386	0.074	0.000	0.251	0.542	*
B3_GLUCOS	0.468	0.091	0.000	0.303	0.663	*
PLACENTA BY						
B1_PLGF_L	0.849	0.075	0.000	0.699	0.984	*
B2_PLGF_L	0.693	0.066	0.000	0.587	0.839	*
INFLAM2 BY						
B1_IL6_LO	0.629	0.059	0.000	0.520	0.752	*
B2_IL6_LO	0.570	0.054	0.000	0.466	0.678	*
SES BY						
INCOME_ENT	0.812	0.040	0.000	0.735	0.894	*
HIGHESTEDU	0.568	0.033	0.000	0.504	0.631	*
IMD_1TO5	-0.361	0.031	0.000	-0.421	-0.300	*
GLYCEMIA ON						
OBESITY	0.177	0.061	0.002	0.058	0.298	*
SES	-0.037	0.075	0.312	-0.186	0.110	
INFLAM2 ON						
OBESITY	0.391	0.044	0.000	0.304	0.475	*
PLACENTA ON						
DEPRESSION	-0.056	0.045	0.105	-0.147	0.031	
INFLAM2	-0.019	0.059	0.374	-0.136	0.095	
OBESITY	-0.073	0.046	0.052	-0.163	0.015	
GLYCEMIA	0.089	0.073	0.107	-0.051	0.237	
SES	-0.207	0.047	0.000	-0.299	-0.116	*
OBESITY ON						
SES	-0.119	0.034	0.000	-0.185	-0.053	*

DEPRESSION ON						
SES	-0.181	0.038	0.000	-0.254	-0.106	*
GA_DEL ON						
OBESITY	0.000	0.000	1.000	0.000	0.000	
DEPRESSION	-0.057	0.036	0.058	-0.125	0.014	
GLYCEMIA	-0.222	0.064	0.000	-0.353	-0.100	*
INFLAM2	-0.031	0.044	0.239	-0.117	0.056	
PLACENTA	-0.039	0.040	0.167	-0.115	0.042	
SES	0.049	0.038	0.098	-0.026	0.124	
TOT3YEPDS ON						
DEPRESSION	0.581	0.038	0.000	0.501	0.651	*
SES	-0.016	0.052	0.380	-0.118	0.087	
SDQHIGH ON						
GLYCEMIA	-0.305	0.201	0.070	-0.681	0.103	
DEPRESSION	0.262	0.103	0.006	0.056	0.461	*
OBESITY	-0.035	0.104	0.367	-0.239	0.168	
INFLAM2	0.001	0.135	0.497	-0.266	0.262	
PLACENTA	-0.292	0.093	0.002	-0.465	-0.103	*
SES	-0.365	0.099	0.000	-0.551	-0.165	*
SDQHIGH ON						
GA_DEL	-0.052	0.046	0.127	-0.143	0.039	
TOT3YEPDS	0.007	0.086	0.466	-0.162	0.174	
F7_NEO_SE	-0.139	0.071	0.027	-0.276	0.003	
INFLAM2 WITH						
DEPRESSION	0.000	0.000	1.000	0.000	0.000	
GLYCEMIA	0.000	0.000	1.000	0.000	0.000	
GLYCEMIA WITH						
DEPRESSION	0.105	0.074	0.073	-0.037	0.251	
OBESITY WITH						
DEPRESSION	0.000	0.000	1.000	0.000	0.000	
Intercepts						
F1_EPDSTO	1.452	0.041	0.000	1.372	1.534	*
F2_EPDSTO	1.283	0.040	0.000	1.204	1.362	*
F3_EPDSTO	1.244	0.041	0.000	1.163	1.325	*
GA_DEL	27.400	0.529	0.000	26.369	28.442	*
B1_GLUCOS	5.942	0.141	0.000	5.669	6.220	*
B3_GLUCOS	4.534	0.124	0.000	4.294	4.779	*
B1_IL6_LO	1.752	0.053	0.000	1.649	1.856	*
B2_IL6_LO	1.246	0.046	0.000	1.156	1.336	*
BMI	7.621	0.149	0.000	7.330	7.917	*
SF_SU	4.474	0.091	0.000	4.297	4.653	*
WAIST	10.136	0.198	0.000	9.751	10.526	*
HIP_CI	11.925	0.232	0.000	11.474	12.384	*
B1_PLGF_L	6.802	0.159	0.000	6.492	7.116	*
B2_PLGF_L	8.280	0.199	0.000	7.893	8.672	*

IMD_1TO5	3.763	0.077	0.000	3.612	3.916	*
HIGHESTEDU	2.006	0.047	0.000	1.914	2.098	*
INCOME_ENT	2.065	0.052	0.000	1.963	2.168	*
TOT3YEPDS	1.267	0.059	0.000	1.152	1.384	*
**Thresholds**						
SDQHIGH$1	-0.682	1.270	0.294	-3.151	1.853	
**Variances**						
SES	1.000	0.000	0.000	1.000	1.000	
**Residual Variances**						
F1_EPDSTO	0.488	0.026	0.000	0.437	0.541	*
F2_EPDSTO	0.199	0.025	0.000	0.152	0.249	*
F3_EPDSTO	0.326	0.026	0.000	0.277	0.380	*
GA_DEL	0.930	0.029	0.000	0.858	0.971	*
B1_GLUCOS	0.851	0.060	0.000	0.707	0.937	*
B3_GLUCOS	0.781	0.090	0.000	0.560	0.908	*
B1_IL6_LO	0.604	0.075	0.000	0.434	0.729	*
B2_IL6_LO	0.675	0.062	0.000	0.540	0.783	*
BMI	0.035	0.014	0.000	0.008	0.063	*
SF_SU	0.711	0.022	0.000	0.668	0.753	*
WAIST	0.494	0.021	0.000	0.454	0.536	*
HIP_CI	0.306	0.017	0.000	0.274	0.340	*
B1_PLGF_L	0.279	0.126	0.000	0.031	0.512	*
B2_PLGF_L	0.520	0.094	0.000	0.297	0.655	*
IMD_1TO5	0.869	0.022	0.000	0.822	0.910	*
HIGHESTEDU	0.677	0.037	0.000	0.602	0.746	*
INCOME_ENT	0.341	0.065	0.000	0.201	0.459	*
TOT3YEPDS	0.657	0.042	0.000	0.576	0.739	*
DEPRESSION	0.967	0.014	0.000	0.936	0.989	*
OBESITY	0.986	0.008	0.000	0.966	0.997	*
GLYCEMIA	0.960	0.025	0.000	0.898	0.993	*
PLACENTA	0.939	0.025	0.000	0.879	0.976	*
INFLAM2	0.847	0.034	0.000	0.775	0.907	*

R-SQUARE

Variable	Estimate	Posterior S.D.	One-Tailed P-Value	95% C.I. Lower 2.5%	95% C.I. Upper 2.5%
SDQHIGH	0.445	0.129	0.000	0.248	0.758
F1_EPDSTOTAL	0.512	0.026	0.000	0.459	0.563
F2_EPDSTOTAL	0.801	0.025	0.000	0.751	0.848
F3_EPDSTOTAL	0.674	0.026	0.000	0.620	0.723
GA_DEL_DAY	0.070	0.029	0.000	0.029	0.142
B1_GLUCOSE	0.149	0.060	0.000	0.063	0.293
B3_GLUCOSE	0.219	0.090	0.000	0.092	0.440
B1_IL6_LOG2	0.396	0.075	0.000	0.271	0.566
B2_IL6_LOG2	0.325	0.062	0.000	0.217	0.460
BMI	0.965	0.014	0.000	0.937	0.992
SF_SUM	0.289	0.022	0.000	0.247	0.332
WAIST	0.506	0.021	0.000	0.464	0.546

HIP_CIRCUM	0.694	0.017	0.000	0.660	0.726
B1_PLGF_LOG2	0.721	0.126	0.000	0.488	0.969
B2_PLGF_LOG2	0.480	0.094	0.000	0.345	0.703
IMD_1TO5	0.131	0.022	0.000	0.090	0.178
HIGHESTEDU	0.323	0.037	0.000	0.254	0.398
INCOME_ENTRY	0.659	0.065	0.000	0.541	0.799
TOT3YEPDS	0.343	0.042	0.000	0.261	0.424

		Posterior	One-Tailed	95% C.I.	
Variable	Estimate	S.D.	P-Value	Lower 2.5%	Upper 2.5%
DEPRESSION	0.033	0.014	0.000	0.011	0.064
OBESITY	0.014	0.008	0.000	0.003	0.034
GLYCEMIA	0.040	0.025	0.000	0.007	0.102
PLACENTA	0.061	0.025	0.000	0.024	0.121
INFLAM2	0.153	0.034	0.000	0.093	0.225

References

[1] Muthén B. Bayesian Analysis In Mplus: A Brief Introduction 2010.

[2] Fuente J de la, Santiago J, Román A, Dumitrache C, Casasanto D. When you think about it, your past is in front of you: how culture shapes spatial conceptions of time. Psychological Science 2014;25:1682–90. doi:10.1177/0956797614534695.

[3] Link WA, Eaton MJ. On thinning of chains in MCMC. Methods in Ecology and Evolution 2012;3:112–5. doi:10.1111/J.2041-210X.2011.00131.X.

[4] Schoot R van de, Kaplan D, Denissen J, Asendorpf JB, Neyer FJ, Aken MAG van. A Gentle Introduction to Bayesian Analysis: Applications to Developmental Research. Child Development 2014;85:842–60. doi:10.1111/cdev.12169.

[5] Akaike H. Factor analysis and AIC. Psychometrika 1987;52:317–32. doi:10.1007/BF02294359.

[6] Nylund KL, Asparouhov T, Muthén BO. Deciding on the Number of Classes in Latent Class Analysis and Growth Mixture Modeling: A Monte Carlo Simulation Study. Structural Equation Modeling: A Multidisciplinary Journal 2007;14:535–69. doi:10.1080/10705510701575396.

[7] Lo Y. Testing the number of components in a normal mixture. Biometrika 2001;88:767–78. doi:10.1093/biomet/88.3.767.

[8] Celeux G, Soromenho G. An entropy criterion for assessing the number of clusters in a mixture model. Journal of Classification 1996;13:195–212. doi:10.1007/BF01246098.

[9] Asparouhov T, Muthén B. Auxiliary Variables in Mixture Modeling: Three-Step Approaches Using M plus. Structural Equation Modeling: A Multidisciplinary Journal 2014;21:329–41. doi:10.1080/10705511.2014.915181.

[10] Wickrama K, Lee TK, O'Neal CW, Lorenz F. Higher-Order Growth Curves and Mixture Modeling with Mplus. Routledge; 2016. doi:10.4324/9781315642741.

[11] Asparouhov T, Muthén B. Bayesian Analysis of Latent Variable Models using Mplus. 2021.

[12] Depaoli S, Schoot R van de. Improving transparency and replication in Bayesian statistics: The WAMBS-Checklist. Psychological Methods 2017;22:240–61. doi:10.1037/met0000065.
